# Identifying Potential Mechanisms Enabling Acidophily in the Ammonia-Oxidizing Archaeon “Candidatus Nitrosotalea devanaterra”

**DOI:** 10.1128/AEM.04031-15

**Published:** 2016-04-18

**Authors:** Laura E. Lehtovirta-Morley, Luis A. Sayavedra-Soto, Nicolas Gallois, Stefan Schouten, Lisa Y. Stein, James I. Prosser, Graeme W. Nicol

**Affiliations:** a Institute of Biological and Environmental Sciences, University of Aberdeen, Aberdeen, United Kingdom; b Department of Botany and Plant Pathology, Oregon State University, Corvallis, Oregon, USA; c Université Blaise Pascal, Clermont-Ferrand, France; d NIOZ Royal Netherlands Institute for Sea Research, Department of Marine Organic Biogeochemistry, and Utrecht University, Den Burg, Texel, The Netherlands; e Department of Biological Sciences, University of Alberta, Edmonton, Alberta, Canada; f Laboratoire Ampère, École Centrale de Lyon, L'Université de Lyon, Ecully, France; Michigan State University

## Abstract

Ammonia oxidation is the first and rate-limiting step in nitrification and is dominated by two distinct groups of microorganisms in soil: ammonia-oxidizing archaea (AOA) and ammonia-oxidizing bacteria (AOB). AOA are often more abundant than AOB and dominate activity in acid soils. The mechanism of ammonia oxidation under acidic conditions has been a long-standing paradox. While high rates of ammonia oxidation are frequently measured in acid soils, cultivated ammonia oxidizers grew only at near-neutral pH when grown in standard laboratory culture. Although a number of mechanisms have been demonstrated to enable neutrophilic AOB growth at low pH in the laboratory, these have not been demonstrated in soil, and the recent cultivation of the obligately acidophilic ammonia oxidizer “Candidatus Nitrosotalea devanaterra” provides a more parsimonious explanation for the observed high rates of activity. Analysis of the sequenced genome, transcriptional activity, and lipid content of “*Ca*. Nitrosotalea devanaterra” reveals that previously proposed mechanisms used by AOB for growth at low pH are not essential for archaeal ammonia oxidation in acidic environments. Instead, the genome indicates that “*Ca*. Nitrosotalea devanaterra” contains genes encoding both a predicted high-affinity substrate acquisition system and potential pH homeostasis mechanisms absent in neutrophilic AOA. Analysis of mRNA revealed that candidate genes encoding the proposed homeostasis mechanisms were all expressed during acidophilic growth, and lipid profiling by high-performance liquid chromatography–mass spectrometry (HPLC-MS) demonstrated that the membrane lipids of “*Ca*. Nitrosotalea devanaterra” were not dominated by crenarchaeol, as found in neutrophilic AOA. This study for the first time describes a genome of an obligately acidophilic ammonia oxidizer and identifies potential mechanisms enabling this unique phenotype for future biochemical characterization.

## INTRODUCTION

Ammonia oxidation is integral to the global nitrogen cycle and is performed by ammonia-oxidizing bacteria (AOB) and ammonia-oxidizing archaea (AOA). AOA are among the most abundant phyla on Earth (1) and frequently outnumber AOB in the environment (2). Ammonia concentration (3) and soil pH (4) have been suggested as drivers of niche differentiation in terrestrial ammonia oxidizers. The effect of soil pH on ammonia oxidation is of particular interest since gross nitrification rates in acidic soils (pH of <5.5), which represent ∼50% of Earth's potentially arable land, are similar to those in neutral soils (5). Until recently, growth of all cultivated prokaryotic ammonia oxidizers in batch culture was considered possible only at a pH of >6.5. This view was challenged by the initial cultivation (6) and subsequent isolation (7) of the obligately acidophilic AOA “Candidatus Nitrosotalea devanaterra,” which grows in the pH range 4.0 to 5.5. Members of the Nitrosotalea lineage are abundant and widely distributed in acidic soils globally (4).

Inhibition of ammonia oxidizer growth and activity at low pH is poorly understood, and several mechanisms have been suggested, including substrate limitation and product toxicity. Kinetic studies of ammonia oxidation in cell suspensions and extracts of Nitrosomonas europaea suggest that ammonia (NH_3_), rather than ammonium (NH_4_^+^), is the substrate for ammonia monooxygenase (AMO), which catalyzes the first step in ammonia oxidation (8). In contrast, the preferred substrate (NH_3_/NH_4_^+^) of AOA has not been identified. The pK_a_ for NH_4_^+^ is 9.25, and ammonia availability decreases with pH (e.g., NH_3_/NH_4_^+^ ratios are 1:200 and 1:20,000 at pH 7 and 5, respectively). Growth on NH_3_, rather than NH_4_^+^, is also supported by lack of growth of AOB in liquid culture below pH 6.5. Although AOB growth occurs under acid conditions in culture (e.g., growth of ureolytic strains on urea [9] or within biofilms [10] or aggregates [11]), there is little evidence that these mechanisms enable AOA growth in acidic soils, nor do they explain acidophilic growth of “*Ca*. Nitrosotalea devanaterra” in laboratory culture.

All known bacterial and archaeal ammonia oxidizers are autotrophs (12, 13). AOB fix CO_2_ via the Calvin cycle (12), whereas AOA fix HCO_3_^−^ via the hydroxypropionate/hydroxybutyrate pathway (13). Most inorganic carbon exists as CO_2_ at low pH, and the low HCO_3_^−^ concentration may require additional adaptations in “*Ca*. Nitrosotalea devanaterra.”

In addition to low NH_3_ and HCO_3_^−^ availability, acidic pH presents a further challenge to cellular pH homeostasis. Almost all known prokaryotic acidophiles maintain a cytoplasmic pH higher than that in the extracellular environment (14). Extreme acidophiles usually have a reverse membrane potential (inside positive), and the proton motive force is constituted by the transmembrane pH gradient. There are several mechanisms of pH adaptation in acidophiles. In some, reverse membrane potential is achieved by uptake of cations (typically potassium), and most acidophile genomes encode a high number of cation transporters (15). The entry of protons is prevented by altering the membrane composition or modifying the cell surface structures (16, 17). Some acidophiles have proton pumps coupled to the electron transfer chain that actively remove protons from the cytoplasm (18). In addition, protons can be scavenged by buffering, e.g., by carbonic anhydrase in Helicobacter pylori (19), or metabolism, e.g., by arginine- and glutamate decarboxylase-based acid resistance mechanisms (20). Since small organic acids can function as uncouplers by passing through the membrane before releasing protons and acidifying the neutral pH cytoplasm, most extreme acidophiles are heterotrophs (15, 21). Acidophiles typically contain a large proportion of secondary transporters coupled to ion gradients (22). In addition, DNA and protein repair genes are prevalent in extreme acidophile genomes and are responsible for the rapid repair required after damage by low pH (15).

“*Ca*. Nitrosotalea devanaterra” is able to grow and oxidize ammonia within the pH range 4 to 5.5 and is unable to grow at neutral pH (6). This physiology makes “*Ca*. Nitrosotalea devanaterra” unique among all bacterial and archaeal ammonia oxidizers and indicates adaptations distinct from those of other cultured ammonia oxidizers. There are several major unresolved questions regarding acidophilic ammonia oxidation, as follows. (i) How does “*Ca*. Nitrosotalea devanaterra” overcome low NH_3_ concentrations? Specifically, can this organism utilize NH_4_^+^, is the active site of AMO facing the cytoplasm or the periplasm and are there other N metabolism genes that can explain the distinct physiology of “*Ca*. Nitrosotalea devanaterra”? (ii) How does “*Ca*. Nitrosotalea devanaterra” fix carbon under acidic conditions where the HCO_3_^−^ concentration is low? (iii) Is cytoplasmic pH homeostasis of “*Ca*. Nitrosotalea devanaterra” achieved by mechanisms similar to those in other acidophiles? The aim of this study was therefore to examine the “*Ca*. Nitrosotalea devanaterra” genome for evidence of specific adaptations in N and C metabolism and to determine whether the genome contained genes involved in pH homeostasis mechanisms found in other model acidophiles.

## MATERIALS AND METHODS

### DNA isolation.

“Candidatus Nitrosotalea devanaterra” Nd1 was grown in pure culture as previously described (7). Biomass from 10 liters of the culture was concentrated using a Pellicon XL tangential-flow filter cassette (pore size, 0.22 μm) (Merck Millipore, Billerica, MA, USA), followed by pelleting of cells by centrifugation (15 min at 18,000 × *g*). High-molecular-weight genomic DNA was extracted from a cell pellet by chemical lysis (23). Briefly, cells were lysed in the presence of proteinase K (final concentration, 100 μg ml^−1^) and SDS (0.5% [wt/vol]), followed by addition of cetyltrimethylammonium bromide (CTAB) in NaCl solution (1% CTAB [wt/vol] and 0.7 M NaCl [final concentrations]) in a final volume of 780 μl. Proteins were removed using an equal volume of phenol-chloroform-isoamyl alcohol (25:24:1) and DNA precipitated in the presence of 7.5 μg linear acrylamide with a 0.7 volume of 100% isopropanol. After centrifugation, the pellet was washed with 70% ethanol and resuspended in Tris-EDTA (TE) buffer. PacBio sequencing was necessary for genome closure, and as larger quantities of DNA (10 μg) were required, approximately 40 liters of culture was harvested by tangential-flow filtration and the extraction was performed using a DNeasy blood and tissue kit (Qiagen, Venlo, Netherlands). A minor modification to the manufacturer's protocol was made: DNA was eluted fives times with 50 μl prewarmed distilled water (dH_2_O) and concentrated using a vacuum concentrator (Eppendorf, Hamburg, Germany). The molecular weight of DNA was assessed by agarose gel electrophoresis, and the concentration was quantified using a NanoDrop spectrophotometer (Thermo Scientific, Wilmington, DE, USA) and a PicoGreen Quant-iT kit (Invitrogen, Carlsbad, CA, USA).

### Genome sequencing, assembly, and deposition.

DNA was sequenced using an Illumina MiSeq sequencer (flow cell v3 with 300-bp paired-end and 2-kb mate pair libraries) and with a PacBio RSII (GTAC, Constance, Germany) that provided 50× and 10× genome coverage, respectively. Assembly of Illumina reads was performed with CLC (CLC bio) and that of PacBio reads with Celera 8.1 (24) and SPAdes 3.1.1 (25). Illumina reads were first quality trimmed, several assembly parameters were tested, and finally the assembly was performed with a word size of 45, a bubble size of 98, and a minimum contig length of 1,000 bp. PacBio reads were error corrected using the PBcR pipeline in Celera and subsequently assembled. Neither approach alone was sufficient to close the genome, and a hybrid assembly of PacBio and Illumina reads was performed with SPAdes. Different assemblies were compared and validated using MAUVE software (26). The entire genome was annotated in MaGe (27) based on a combination of bioinformatics tools, including homology-, structure-, and synteny-based approaches. Other AOA genomes are included in MaGe, which relies on the PkGDB relational database with data from both public data banks and manually curated genomes. Manual curation of the “*Ca*. Nitrosotalea devanaterra” genome was performed in MaGe, with emphasis on genes absent in other AOA, genes located at synteny breaks, genes characterized in other AOA, and functions involved in major metabolic pathways. Functional categorization into clusters of orthologous genes (COGs) and protein families (Pfams) was performed with COGnitor and HMMPfam software implemented in MaGe, respectively. tRNA predictions were made using tRNAscan-SE (28) and ARAGORN (29). Transporters were annotated using TransAAP implemented in TransportDB (30). Average nucleotide identity (ANI) and tetranucleotide frequency analyses were carried out in JSpecies (31). z scores of the nucleotide word frequency analysis were used to build the matrix for the principal-component analysis in PAST software (32) and SigmaPlot v12 (Systat Software Inc., London, United Kingdom). To construct Venn diagrams, the presence of open reading frames (ORFs) in archaeal genomes was identified using reciprocal BLASTP searches (selected cutoffs were an E value of 10^−3^ and 30% amino acid identity) implemented in the stand-alone BLAST-2.2.25+ suite (33). The cutoff for absence and presence of coding sequences (CDS) in comparison with other archaeal and bacterial genomes was selected as 30% DNA identity with 80% CDS coverage, as recommended in the MaGe guidelines. Synteny analysis was performed in MaGe (27).

### Identification of genes potentially linked to acidophily.

To identify candidate genes enabling acidophily, the “*Ca*. Nitrosotalea devanaterra” genome was compared to those of previously characterized archaeal and bacterial acidophiles to detect gene homologues conserved between “*Ca*. Nitrosotalea devanaterra” and acidophiles and absent in other AOA (cutoff, 30% DNA identity with 80% CDS coverage). However, as “*Ca*. Nitrosotalea devanaterra” represents an uncharacterized genus, many ORFs had no database hits in RefSeq, Swiss-Prot, and TrEMBL. Many ORFs also had homology only to uncharacterized proteins in databases. In addition, some (novel) acid tolerance mechanisms may be a result of convergent evolution and not share genetic homology. Therefore, a literature search was performed on pH adaptation strategies of acidophilic microorganisms, as well as on biochemical limitations of ammonia oxidizers with regard to pH, and potential functional analogues were manually identified from the curated “*Ca*. Nitrosotalea devanaterra” genome.

### Lipid analysis.

The intact polar and core lipids of “*Ca*. Nitrosotalea devanaterra” were analyzed as described previously (82). For analysis of intact polar lipids, an aliquot of Bligh-Dyer extracts (BDEs) was analyzed with high-performance liquid chromatography–electrospray ionization–tandem mass spectrometry (HPLC-ESI-MS/MS). For analysis of core glycerol dibiphytanyl glycerol tetraether lipids (GDGTs), acid hydrolysis was performed on aliquots of BDEs to cleave off polar head groups and release core GDGTs, which were analyzed by HPLC-atmospheric pressure chemical ionization-MS (HPLC-APCI-MS) using a modified procedure (34).

### Sequence alignments, protein structure predictions, and phylogenetic analysis.

Sequence alignments of AmoB, AmoC, and Amt/Rh genes were generated using ClustalW implemented in BioEdit (35) and used to examine active-site conservation and for phylogenetic analysis. For AMO, all AOA *amoB* and *amoC* sequences deposited in GenBank (July 2014) were used in alignments, and the active site of AMO was modeled against PmoB and PmoC of Methylococcus capsulatus Bath and other previously characterized model organisms (36–39). This resulted in an alignment of 115 and 147 sequences for *amoB* and *amoC*, respectively. Transmembrane helices and protein topology were predicted initially using TmHMM (40). However, TmHMM failed to detect some of the helices that experimentally have been indicated to be present in AOA AmoB sequences (39), and TMPred (41) and TopPred (42) were subsequently used. Signal peptides were initially predicted by SignalP (43), which also failed to detect signal peptides of AmoB of AOA (39), and prediction was implemented in Phobius (44). Phobius was used additionally for validation of the transmembrane helix predictions. Phobius uses both signal peptides and transmembrane helices in its topology prediction, which requires that amino acid residues immediately downstream from the signal peptide cleavage site must be extracellular.

For Amt/Rh, the database was compiled from a selected set of sequences (total, 89) from GenBank, with the emphasis on sequences that had been previously characterized electrophysiologically and structurally. Phylogenetic analysis of derived Amt/Rh protein sequences used 228 unambiguously aligned positions. Maximum-likelihood analysis was performed with PhyML (45) with invariable sites and eight variable gamma rates modeled. Bootstrap support was also calculated using parsimony and distance analyses (MEGA6 [46]) with 1,000 replicates.

### Transcriptional and physiological response to pH change.

Exponentially growing “*Ca*. Nitrosotalea devanaterra” cultures (pH 5) were placed into prerinsed and autoclaved dialysis bags (Spectra/Por 4, molecular mass cutoff, 12 to 14 kDa [Spectrum Labs, Rancho Dominguez, CA, USA]) and transferred twice to fresh medium (pH 5) to remove nitrite to avoid inhibition before transfer to media at pH 4.0, 5.0, and 6.0. The pH was recorded at the start and end of the experiments, and mean pH values were 4.2, 5.5, and 6.2 and 4.2, 5.6, and 6.5, respectively. At each time point, three replicate dialysis bags were destructively sampled. Nitrite was measured inside and outside the bag, and 1 ml of cells was used for transcriptional analysis. Cell and *amoA* abundances were estimated at all time points for all replicates. Phenol-ethanol (1:19) stop solution was added immediately after sampling to preserve transcripts before storing at −80°C until RNA extraction. Nucleic acid extraction was performed by bead beating in the presence of buffer containing 0.5% Triton-X, 0.4% *N*-lauroylsarcosine, 0.4% SDS, 50 mM Tris-HCl (pH 8), and 100 mM EDTA in a total volume of 500 μl, followed by treatment with an equal volume of phenol-chloroform-isoamyl alcohol (25:24:1). Nucleic acids were precipitated in the presence of 7.5 μg linear acrylamide, 0.1 volume 3 M sodium acetate, and an equal volume of 100% ethanol, followed by DNase treatment and reverse transcription (47). Primers were designed for selected transporters and putative pH homeostasis genes using Primer3 (see Table S1 in the supplemental material) (48), and quantitative PCR (qPCR) was performed with QuantiFast Sybr green mix (Qiagen) for all the assays using a two-step cycle: initial denaturation at 95°C for 15 min, followed by 35 cycles of denaturation at 95°C for 10 s, combined annealing/extension at 60°C for 30 s, and fluorescence reading at 72°C, followed by a final extension at 72°C for 10 min, followed by the melt curve from 55°C to 95°C. The quality of qPCR products was verified by melt curve analysis and agarose gel electrophoresis. *amoA* qPCR was performed as previously described (6). Statistical analysis was performed in SigmaPlot v12 (Systat Software Inc., London, United Kingdom). To determine whether there was a significant increase or decrease in transcript abundance over time, linear regression was performed using individual (rather than averaged) values of transcript/gene data as the dependent variable and time as the independent variable. This analysis was additionally performed for nitrite accumulation, assessed with Griess reagent as previously described (7).

### Nucleotide sequence accession number.

The closed genome sequence with full annotations has been deposited in ENA with accession number LN890280.

## RESULTS AND DISCUSSION

### Genome summary.

The closed and complete genome of “*Ca*. Nitrosotalea devanaterra” is a single circular 1,805,304-bp chromosome with 2,205 predicted genes (Table 1). The genome was automatically annotated prior to manual examination and curation, and a putative function was successfully assigned to 58.8% of ORFs and seven small RNA (smRNA) genes, 40 tRNA genes, and 1 copy of each rRNA gene (5S, 16S, and 23S), with putative introns in various tRNA sequences and the 23S rRNA gene. Tetranucleotide frequency analysis indicates that the “*Ca*. Nitrosotalea devanaterra” genome is distinct from those of other AOA genera and that ∼26% of ORFs of “*Ca*. Nitrosotalea devanaterra” are absent from other soil AOA genomes (see Fig. S1 in the supplemental material). Approximately 15% (364) of ORFs of “*Ca*. Nitrosotalea devanaterra” are unique when all aquatic AOA are included in this comparison. The distinction of genus Nitrosotalea from other AOA is supported by the low average nucleotide identity (ANI) scores between “*Ca*. Nitrosotalea devanaterra” and group 1.1a and 1.1b representatives (66.5 to 68.0% and 62.5 to 62.9%, respectively) (see Fig. S1 in the supplemental material).

**TABLE 1:** Genomic features of selected thaumarchaea

Parameter	Value				
	“*Ca*. Nitrosotalea devanaterra” Nd1	“*Ca*. Nitrosopumilus koreensis” MY1	“*Ca*. Nitrosopumilus maritimus” SCM1	“*Ca*. Nitrososphaera viennensis” EN76	“*Ca*. Nitrososphaera gargensis” Ga9.2
Length (Mb)	1.81	1.61	1.65	2.53	2.83
Coding density (%)	92.1	89.99	91.7	87.0	83.4
No. of predicted CDS	2,205	1,957	1,967	3,123	3,997
% of annotated CDS	58.8	62.1	56.0	46.7	43.1
No. of:					
16-23S rRNA operons	1	1	1	1	1
Separate 5S rRNAs	1	1	1	1	1
tRNAs	40	42	44	44	40
Other RNAs	7	3	1	5	0
Primary transporter genes	51	38	45	66	59
Secondary transporter genes	42	41	31	44	54
Other transporter genes	14	14	18	22	26
Ratio of secondary to primary transporter genes^*a*^	0.8:1	1.1:1	0.7:1	0.7:1	0.9:1

a Ratios of secondary to primary transporter genes in other archaeal acidophiles (41): Thermoplasma acidophilum, 10:1; Picrophilus torridus, 5.6:1; Sulfolobus solfataricus, 2.7:1.

“*Ca*. Nitrosotalea devanaterra” has major metabolic pathways that are conserved in all AOA and discussed elsewhere (49, 50), including biosynthesis of amino acids, lipids, and sugars and central C metabolism (see Table S2 in the supplemental material). In addition, “*Ca*. Nitrosotalea devanaterra” contains an Ni-Fe hydrogenase and genes for flagellar motility, gas vacuoles, and phosphorus utilization, which are present in some but not all AOA (see Tables S2 and S3 in the supplemental material). It is, however, beyond the scope of this article to discuss all of these genes individually, and this article highlights those features that could be associated with the adaptation of “*Ca*. Nitrosotalea devanaterra” to low-pH environments.

### Similarities between “*Ca*. Nitrosotalea devanaterra” and other acidophiles.

The fully annotated “*Ca*. Nitrosotalea devanaterra” genome contains 364 ORFs without homologues in neutrophilic AOA genomes, of which 45 were shared with characterized bacterial and archaeal acidophiles (see Table S4 in the supplemental material). No homologues were found exclusively in all acidophiles, and only few were present in most acidophiles: Na^+^/solute symporter (NDEV_1297), 2 major facilitator superfamily (MFS) transporters (NDEV_1231 and NDEV_1448), 2 natural resistance-associated macrophage protein (NRAMP) divalent cation transporters (NDEV_1079 and NDEV_1085), an FK506 binding protein (FKBP)-type peptidyl-prolyl *cis-trans* isomerase (NDEV_0529), conserved hypothetical proteins (NDEV_0373 and NDEV_1669), 2 transcriptional regulators (NDEV_0570 and NDEV_1462), and several subunits of archaeal A-type ATP synthase (complex V) which correspond to the membrane-bound A_0_ domain and the central stalk (NDEV_1999 [*atpI*], NDEV_2002 [*atpD*], NDEV_2005 [*atpF*], and NDEV_2006 [*atpC*]) (51) (see Table S5 in the supplemental material). Of these, cation transporters may function in cation uptake, ATP synthase may function in proton translocation, and FKBP-type peptidyl-prolyl *cis-trans* isomerase may facilitate protein folding. This modest conservation probably reflects the fact that bacterial and archaeal acidophiles have several strategies for pH adaptation (15). In addition, “*Ca*. Nitrosotalea devanaterra” may possess mechanisms enabling acidophily that are distinct from those of other acidophiles. The genome, with the emphasis on genes absent in other AOA, was therefore further investigated to identify potential mechanisms contributing to pH homeostasis and facilitating growth of AOA with low concentrations of NH_3_ and HCO_3_^−^.

### Ion transport.

**FIG 1:**
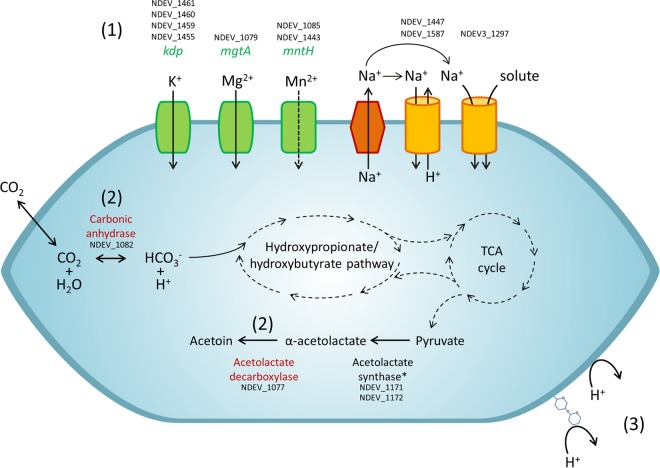
Predicted mechanisms of cytoplasmic pH regulation in “*Ca*. Nitrosotalea devanaterra” based on the presence of putative functional genes in the genome. (1) Cation influx and proton efflux. *kdp*, potassium transporting P-type ATPase gene; *mgtA*, putative magnesium-transporting P-type ATPase gene; *mntA*, NRAMP-type divalent cation transporter (two copies) gene, Na^+^/solute transporter, Na^+^/hydrogen exchanger (two copies). (2) Proton consumption by metabolism: acetolactate decarboxylase, carbonic anhydrase. (3) Reduced permeability of the cell wall/cell membrane: cell surface glycosylation, GDGT-4-dominated membrane.

Extreme acidophiles contain a large number of secondary transporters (22), but “*Ca*. Nitrosotalea devanaterra,” surprisingly, possesses a similar number of secondary transporters to the number possessed by other AOA (Table 1; see Table S5 in the supplemental material) and has proportionally more primary transporters than secondary transporters. The genome of “*Ca*. Nitrosotalea devanaterra” contains a variety of cation transporters, with predicted uptake of K^+^, Na^+^, and divalent cations (Fig. 1). Na^+^/solute symporter (NDEV_1297) is absent in other AOA and is one of the few genes consistently found across acidophilic archaeal genomes (see Table S4 in the supplemental material). “*Ca*. Nitrosotalea devanaterra” lacks the typical CPA2 family Na^+^/H^+^ exchangers of other AOA and has two CPA1 family Na^+^/H^+^ exchangers (NDEV_1447 and NDEV_1587), which share low identity (19.8%) (see Table S6 in the supplemental material). “*Ca*. Nitrosotalea devanaterra” has two NRAMP family transporters (NDEV_1085 and NDEV_1443) that are absent in other AOA and which may transport divalent cations, e.g., Mn^2+^, Fe^2+^, Zn^2+^, Cd^2+^, and Co^2+^.

**FIG 2:**
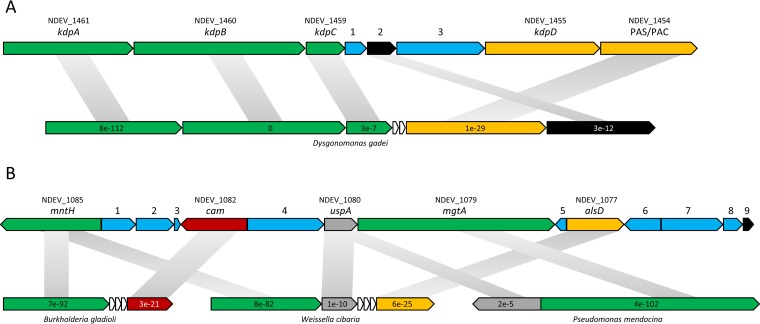
Gene organization within potassium transporter (A) and pH homeostasis (B) gene clusters. Arrows indicate the relative lengths and directions of ORFs. Values inside the arrows indicate synteny E values. Values above the arrows indicate the gene name. (A) *kdpABC*, potassium-transporting ATPase gene; *kdpD*, signal transduction histidine kinase gene; PAC/PAS, PAS/PAC sensor histidine kinase gene; 1, gene for protein of unknown function; 2, signal transduction response regulator gene; 3, peptidase gene. (B) *mntH*, magnesium/divalent cation transporter gene; *cam*, carbonic anhydrase gene; *uspA*, universal stress protein gene; *mgtA*, magnesium transporting P-type ATPase gene; *alsD*, putative alpha-acetolactate decarboxylase gene; 1, phosphoribosyltransferase-like protein gene; 2, gene for conserved protein of unknown function; 3, Val tRNA gene; 4, adenylate/guanylate cyclase sensor protein gene; 5, gene for protein of unknown function; 6, response regulator receiver protein gene; 7, secreted copper domain-containing protein gene; 8, putative adenylate cyclase gene; 9, putative transposase gene.

The genome carries genes encoding two P-type ATPases, *kdpABCD* (NDEV_1461, NDEV_1460, NDEV_1459, and NDEV_1455) and *mgtA* (NDEV_1079), predicted for K^+^ and Mg^2+^ transport, respectively (Fig. 1 and 2). While *mgtA* is not found in AOA except “*Ca*. Nitrosotalea devanaterra” (see Table S4 in the supplemental material), the *kdp* gene cluster is syntenic in many prokaryotes, e.g., proteobacteria, Nitrososphaera viennensis, and acidophilic archaea (Fig. 2A). “*Ca*. Nitrosotalea devanaterra” also lacks the *trk* potassium transporter gene conserved in many AOA genomes (see Table S6 in the supplemental material). Potassium is typically a critically important solute for maintaining the reverse membrane potential in acidophiles (15), and it is therefore surprising that the “*Ca*. Nitrosotalea devanaterra” genome does not contain genes for potassium transporters which are absent in all neutrophilic AOA.

Several of the “*Ca*. Nitrosotalea devanaterra” genes potentially involved in pH/ion homeostasis are colocalized on the chromosome (*mgtA*, *mntH*, a carbonic anhydrase gene [NDEV_1082], a universal stress protein gene [NDEV_1080], and an α-acetolactate decarboxylase gene [NDEV_1077]). Interestingly, the region is flanked by a transposase gene, located at a synteny break, and the gene cluster shares a degree of synteny and homology with bacterial genomes (Fig. 2B; see Table S7 in the supplemental material).

### Scavenging protons by metabolism.

Several bacterial acid resistance mechanisms are based on cytoplasmic proton consumption by various decarboxylases (20). All AOA contain an arginine decarboxylase (NDEV_1165) that is involved in arginine and polyamine metabolism but also in arginine-based acid resistance in bacteria. Due to its presence in neutrophilic AOA, it is an unlikely candidate for pH adaptation of “*Ca*. Nitrosotalea devanaterra.” In contrast to neutrophilic AOA, “*Ca*. Nitrosotalea devanaterra” harbors a homologue of α-acetolactate decarboxylase (NDEV_1077), which is upregulated in bacteria during pH stress (52) and is predicted to catalyze the conversion of α-acetolactate to acetoin (Fig. 1 and 2). This is unexpected, as acetoin production is energetically costly for an autotrophic organism and α-acetolactate decarboxylase activity is generally greater under anaerobic conditions (53). However, α-acetolactate is produced in all AOA from pyruvate as part of valine, leucine, and isoleucine biosynthesis.

### Cell wall and membrane.

The membranes of some acidophiles, e.g., Picrophilus oshimae, require a low pH for stability (16), and the lipid composition and proportion of glycolipids change with varying pH (17). “*Ca*. Nitrosotalea devanaterra” contains two large cell surface modification gene clusters (31 and 34 ORFs) that are absent in neutrophilic AOA, with one cluster located next to genes encoding the main S-layer protein. These genes share only modest homology (30 to 61.4% amino acid identity) with bacterial and a limited number of archaeal genes. However, COG- and Pfam-based functional classification and their synteny with bacterial genomes support their involvement in cell surface glycosylation (see Table S8 and Fig. S2 in the supplemental material). The gene clusters of “*Ca*. Nitrosotalea devanaterra” do not belong to the recognized archaeal S-layer glycosylation pathways of Euryarchaeota and Crenarchaeota (54, 55). In bacteria, these homologues function in synthesis of the polysialic acid capsule (*neu/kps*), pseudoaminic acid glycosylation of flagella (*pse*), and outer spore coat glycosylation of Gram-positive bacteria (*sps*) (but not in bacterial peptidoglycan synthesis or modification) (56–58). There is also no evidence for the presence of peptidoglycan or pseudopeptidoglycan in “*Ca*. Nitrosotalea devanaterra,” which encodes two S-layer proteins (NDEV_0159 and NDEV_0294). It is plausible that the glycosylation genes act by adding nonulosonic acids to the S-layer of “*Ca*. Nitrosotalea devanaterra.”

**FIG 3:**
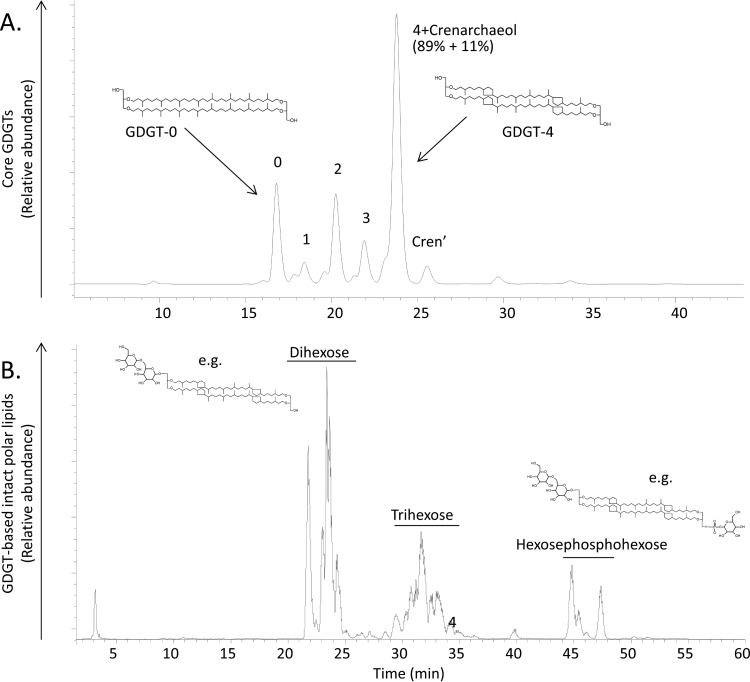
Membrane lipid composition of “*Ca*. Nitrosotalea devanaterra.” Base peak chromatograms of core glycerol dibiphytanyl glycerol tetraether lipids (GDGTs) (A) and GDGT-based intact polar lipids (B) are shown. Cren′ refers to a regioisomer of crenarchaeol. Percentages below “4+Crenarchaeol” indicate the relative contributions of GDGT-4 and crenarchaeol to the peak, respectively. Peaks belong to various core GDGTs from 0 to 4, including GDGTs with hydroxy cores.

The membrane lipid composition of “*Ca*. Nitrosotalea devanaterra” was investigated to determine whether it was distinct from that of neutrophilic AOA and therefore a potential adaptation to low pH. Thaumarchaea have glycerol dibiphytanyl glycerol tetraether lipids (GDGTs), with crenarchaeol typically the dominant GDGT in AOA (accounting for >30% of all lipids [24, 59–61]), but its proportion is much lower (11%) in “*Ca*. Nitrosotalea devanaterra.” Instead, GDGT-4 was the dominant membrane lipid in “*Ca*. Nitrosotalea devanaterra” (Fig. 3). The dominance of GDGT-4 over GDGT-0 to -3 in “*Ca*. Nitrosotalea devanaterra” compared to other AOA is in agreement with culture studies of acidophilic archaea which show an increase in the number of cyclopentane moieties in GDGTs with decreasing pH (62, 63). Furthermore, the increase in rings is thought to increase the packing density of the lipid membrane (64), which may reduce membrane permeability. The head groups of the “*Ca*. Nitrosotalea devanaterra” GDGTs are dominated by sugar moieties (glycolipids), in agreement with previous reports on acidophilic archaea which showed an increase in glycolipids versus phospholipids with decreasing pH (16). An increase in sugar units on the outside cell wall has been suggested to provide protection against proton entry (65). Hydroxylated GDGTs were also detected as cores, as in group 1.1a AOA (60), consistent with the 16S rRNA and *amoA* gene phylogenetic placement of Nitrosotalea (6).

### Central carbon metabolism and acidophily.

“*Ca*. Nitrosotalea devanaterra” possesses a carbonic anhydrase (NDEV_1082) (Fig. 1 and 2), which is present in many acidophiles (see Table S4 in the supplemental material) and predicted to catalyze rapid reversible interconversion of HCO_3_^−^ and CO_2_. Although found also in Nitrososphaera viennensis and “Candidatus Nitrososphaera evergladensis,” carbonic anhydrase may have a dual function for carbon accumulation and cytoplasmic buffering in “*Ca*. Nitrosotalea devanaterra.” Depending on the direction of the reaction, carbonic anhydrase may prevent cytoplasmic acidification by breakdown of HCO_3_^−^ or conversion of membrane-permeative CO_2_ to HCO_3_^−^ for carbon fixation. The reverse mechanism was proposed for carbon assimilation in N. europaea (66), with HCO_3_^−^ transporter and carbonic anhydrase genes colocalized and HCO_3_^−^ taken up, converted to CO_2_, and fixed by the Calvin cycle.

### Ammonia oxidation at low pH.

The closed genome of “*Ca*. Nitrosotalea devanaterra” does not carry any genes participating in N metabolism that could explain its adaptation to apparently low substrate concentrations at acid pH (e.g., with urease). As AMO genes and ammonium transporters are found in all AOA (49), this suggests that the adaptations enabling “*Ca*. Nitrosotalea devanaterra” to grow with low ammonia concentrations either are based on differences from the active sites of AMO and transporters or reflect features shared by all AOA.

**FIG 4:**
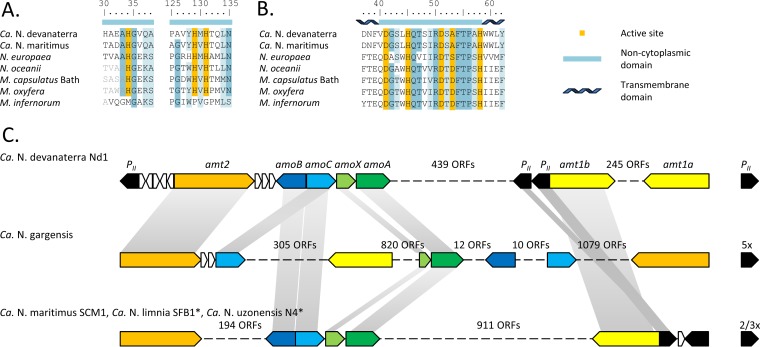
Ammonia oxidation machinery of “*Ca*. Nitrosotalea devanaterra.” (A and B) Conservation of the periplasmic active site in PmoB/AmoB (A) and PmoC/AmoC (B) sequences. (C) AMO gene cluster, Amt transporter, and P_II_ gene arrangement. Longer alignments and protein topology predictions for AmoB and AmoC are in Fig. S3 and S4 in the supplemental material, respectively. Illustrated ORFs are drawn to scale, except for genes that are not involved in ammonia metabolism (truncated, white). The asterisks indicate that the “Candidatus Nitrosoarchaeum limnia” SFB1 and “Candidatus Nitrosotenuis uzonensis” genomes are not closed and distance cannot be estimated.

Since AOB cannot oxidize ammonia at low pH (8, 83), the substrate acquisition systems of “*Ca*. Nitrosotalea devanaterra,” AOA, and AOB were compared. The three histidine residues (His^33^, His^137^, and His^139^ in M. capsulatus Bath [37]) of AmoB/PmoB coordinating a periplasmic dicopper center are conserved in methanotrophs, AOB, and AOA (Fig. 4) but not in the acidophilic methane-oxidizing Verrucomicrobia (36, 37). In contrast, the variable metal binding site of AmoC is present in Verrucomicrobia, AOB, and AOA (38) (Fig. 4). *In silico* protein topology prediction for AmoB and AmoC favors an extracellular (outward-facing) location of the active site in “*Ca*. Nitrosotalea devanaterra” (see Fig. S3 and S4 in the supplemental material). The active site of AMO is conserved between “*Ca*. Nitrosotalea devanaterra,” AOA, and AOB and thus cannot explain the differences between these organisms.

**FIG 5:**
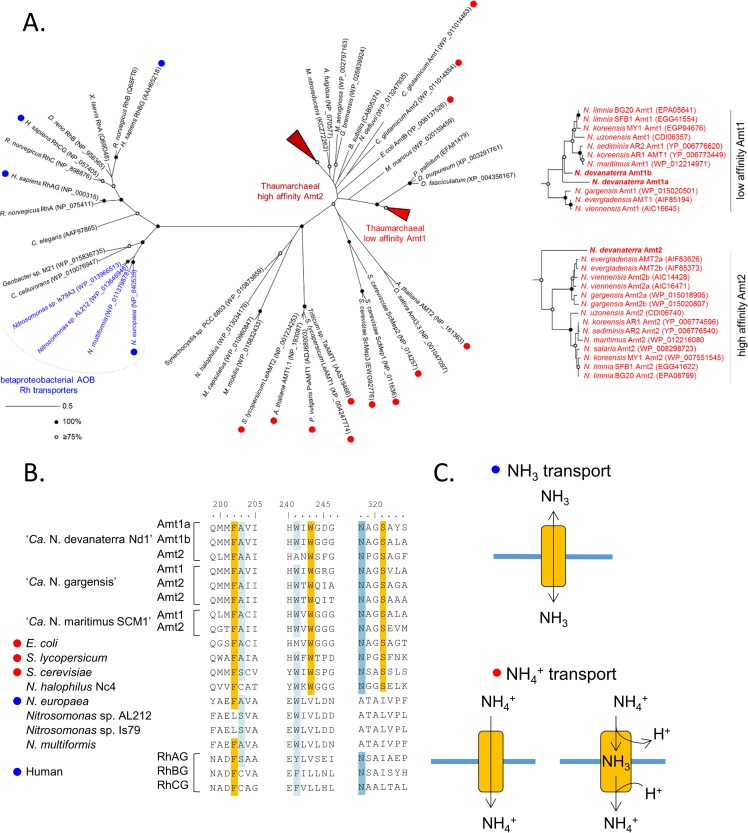
Ammonium transporters of “*Ca*. Nitrosotalea devanaterra.” (A) Maximum-likelihood phylogeny of Amt/Rh transporters. The transported substrate (ammonia versus ammonium) has been validated in organisms highlighted with red and blue circles. Red, ammonium (NH_4_^+^); blue, ammonia (NH_3_). Circles at nodes highlight the level of bootstrap support, and the scale bar represents 0.5 change per amino acid position. (B) Conservation of the cation binding site in Amt/Rh sequences. (C) Transport mechanisms of Amt/Rh transporters previously reported in the literature (see Table S9 in the supplemental material).

Ammonium transporters of AOA and AOB are distantly related and belong to two functionally and phylogenetically distinct families: Amt and Rh, respectively (67). The substrate preferences (NH_3_ versus NH_4_^+^) often differ between Amt and Rh types (68, 69). Rh transporters are thought to perform bidirectional diffusion equilibrating NH_3_ (70), and Amt transporters are energy dependent (71) (Fig. 5C). Furthermore, Amt transporters function better or equally well at acidic pH, whereas Rh transporters favor neutral/high pH (68, 72). Although archaeal ammonium transporters were extensively reviewed recently (67), the transported species (NH_3_ versus NH_4_^+^) of Amt/Rh transporters remains uncertain, and no hypothesis has been put forward for the preferred substrate.

Homology and conservation of coordinating residues with other transporters indicate that NH_4_^+^ is bound to the Amt of “*Ca*. Nitrosotalea devanaterra” (Fig. 5; see Table S9 in the supplemental material). The cation binding site (required for NH_4_^+^, but not NH_3_, transport) is conserved in every Amt of all AOA and is absent in betaproteobacterial AOB (Fig. 5B) (73), as confirmed by the crystal structure of the N. europaea Rh protein (74). In addition, ammonium transporters may play a more critical role in AOA than in AOB, as all AOA genomes contain ≥2 Amt transporters, while five of 10 publically available AOB genomes (N. eutropha C71, Nitrosomas cryotolerans ATCC 49181, Nitrosococcus watsonii C113, Nitrosococcus oceani AFC27, and N. oceani ATC19707) lack ammonia transporters. Unlike neutrophilic AOA, “*Ca*. Nitrosotalea devanaterra” has two putative low-affinity Amts (Amt1a and Amt1b) and one high-affinity Amt (Amt2) with a conserved signal peptide (75). The conservation of the cation binding site suggests that the mechanism enabling “*Ca*. Nitrosotalea devanaterra” to acquire ammonium is shared with all AOA but not AOB.

P_II_ homologues are one of the most widespread signal transduction proteins in prokaryotes and have a vital and exclusive role in regulating N metabolism (76). P_II_ homologues are found in the vicinity of low-affinity Amt genes in neutrophilic group 1.1a AOA (Fig. 4C) (67). In addition, a P_II_ homologue is also located near the high-affinity Amt in “*Ca*. Nitrosotalea devanaterra” (Fig. 4C). Some AOB, e.g., N. europaea, lack P_II_ genes despite the presence of the Rh transporter (66).

### Transcriptional activity of genes potentially involved in pH homeostasis, ammonia acquisition, and ammonia oxidation.

Several genes potentially associated with the acidophilic lifestyle of “*Ca*. Nitrosotalea devanaterra” were chosen for further transcriptional analysis: the genes for potassium-transporting ATPase B chain (*kdpB* [NDEV_1460]), carbonic anhydrase (*cam* NDEV_1082), α-acetolactate decarboxylase (*alsD* [NDEV_1077]), or ammonium acquisition and oxidation (*amt1a* [NDEV_1347], *amt1b* [NDEV_1108], *amt2* [NDEV_1784], and *amoA* [NDEV_1777]). All ammonium transporter genes, *amoA*, and putative pH homeostasis genes, including those absent in neutral-pH AOA, were transcriptionally active. The transcript/gene ratios were highest at the optimal growth pH of 5 (see Fig. S5 and Table S10 in the supplemental material), and transcription rates were generally associated with nitrification activity rather than being in response to pH change. Nevertheless, transcription of ammonium transporter genes, *amoA*, and homeostasis genes at pH 5 highlights their necessity for growth and metabolism in “*Ca*. Nitrosotalea devanaterra.” Transcription of the putative high-affinity Amt2 gene was >10-fold higher than that of the low-affinity Amt1a and Amt1b genes in “*Ca*. Nitrosotalea devanaterra,” as reported for nonstarved neutrophilic AOA in culture and metatranscriptomic studies (75, 77).

### Obligate acidophily.

Representatives of the Nitrosotalea lineage are found overwhelmingly in acidic, rather than neutral, soils (4), and characterized Nitrosotalea isolates cannot grow at a pH of >6.1 (7), but the genome analysis provided no explanation for this inability. Ammonia may be toxic at neutral pH due to its higher concentration and its ability to permeate membranes. Neutral pH could also disable secondary transporters coupled to membrane pH gradients and change the bioavailability of many metals compared to that at acid pH (78).

### How does acidophilic ammonia oxidation occur in “*Ca*. Nitrosotalea devanaterra”?

Several lines of evidence favor an extracellular (outward-facing) orientation of the AMO active site in “*Ca*. Nitrosotalea devanaterra.” First, the active site of the pMMO of M. capsulatus Bath is periplasmic (36), and there is a high degree of conservation across methanotrophs, AOB, and AOA (38). Second, *in silico* topology prediction for AmoB and AmoC of “*Ca*. Nitrosotalea devanaterra” supports the outward orientation of the AMO active site. Third, AMO activity generates reactive intermediates, and it is advantageous to exclude them from the cytoplasm by performing the ammonia oxidation in the pseudoperiplasmic space (79).

**FIG 6:**
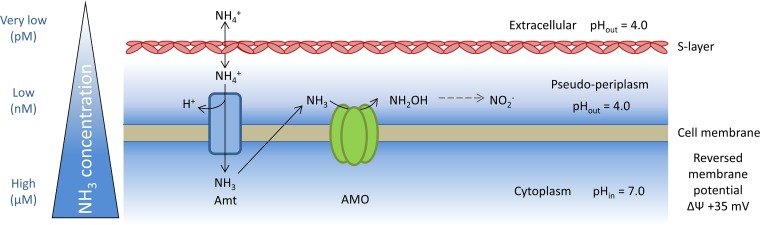
Conceptual model of ammonia acquisition and oxidation mechanisms in “*Ca*. Nitrosotalea devanaterra.” The model is based on references 70 and 81 and work in this study.

Acidophilic growth could be facilitated by high substrate affinity: the whole cells of the marine AOA “Candidatus Nitrosopumilus maritimus” have an apparent half-saturation constant, *K_m_*(NH_3_+NH_4_^+^), of 133 nM at pH 7.5 (2, 80), which is equivalent to ∼2 nM NH_3_ at pH 7.5. At pH 4, 133 nM (NH_3_+NH_4_^+^) is equivalent to ∼0.75 pM, and ammonia oxidation at pH 4 would therefore require a *K_m_*(NH_3_) for AMO in the picomolar range or a mechanism for generating high localized concentrations of ammonia. Import of NH_4_^+^ followed by diffusion of membrane-permeative NH_3_ into the pseudoperiplasm might generate local ammonia concentrations sufficiently high for oxidation (Fig. 6). NH_4_^+^, rather than NH_3_, is abundant at low pH, but oxidation of NH_4_^+^ by the AMO would necessitate substantial changes to the enzyme, which would likely be evident in AMO sequence comparisons.

The regulation of pH consumes energy and may explain the lower growth yields of Nitrosotalea isolates (7) compared to neutral AOA (80). Unlike extreme acidophiles, “*Ca*. Nitrosotalea devanaterra” may not have a reverse membrane potential under all conditions. Assuming the same proton motive force as for N. europaea (−140 mV) (81) and a cytoplasmic pH of 7, membrane potential would be inside positive only at pHs of <4.6 at 25°C (Fig. 6).

The ammonia oxidation machineries of “*Ca*. Nitrosotalea devanaterra” and other AOA are similar, but most cultivated AOA cannot grow at acidic pH. This may be explained by the absence of the cytoplasmic pH regulation mechanisms found in “*Ca*. Nitrosotalea devanaterra,” e.g., ion transport and cell envelope modification, from other AOA. Conversely, even if AOB possessed genes for acid pH homeostasis, starvation would result from a lack of ammonia (without ureolytic activity). If AOA are able to use ammonium for transport as our results suggest, then AOA rather than AOB were in a better predisposition to evolve an acidophilic phenotype.

### Conclusions.

Despite its ecological significance, the mechanism of acid-tolerant ammonia oxidation is a long-standing paradox. This investigation has examined, for the first time, how an ammonia-oxidizing organism may perform this process at low pH by examining an archaeal genome with specific emphasis on (i) N metabolism, (ii) C metabolism, and (iii) pH homeostasis mechanisms. We predict the following. (i) Previously proposed mechanisms of acid tolerance in AOB (e.g., urease, biofilm, or aggregate formation) are not required for the growth of the obligate acidophile “*Ca*. Nitrosotalea devanaterra.” Further, predictions suggest that the AMO of “*Ca*. Nitrosotalea devanaterra” oxidizes NH_3_ and has an extracellular (outward-facing) active site and that Amt transporters of “*Ca*. Nitrosotalea devanaterra” bind NH_4_^+^, in contrast to AOB, which transport NH_3_. (ii) HCO_3_^−^, required for carbon fixation, may be supplied by carbonic anhydrase, which is actively transcribed in “*Ca*. Nitrosotalea devanaterra.” (iii) “*Ca*. Nitrosotalea devanaterra” shares aspects of pH homeostasis mechanisms with other moderate acidophiles, including having a high number of cation transporters, cytoplasmic proton-scavenging mechanisms, and altered cell membrane and surface compositions compared to neutrophilic AOA. In contrast to the case for extreme acidophiles, secondary transporters are not overrepresented in “*Ca*. Nitrosotalea devanaterra.” We postulate that unlike AOB, all AOA, including “*Ca*. Nitrosotalea devanaterra,” have the genetic potential to oxidize ammonia under acidic conditions and that acidophilic adaptation of “*Ca*. Nitrosotalea devanaterra” is achieved by cytoplasmic pH regulation mechanisms not shared by neutrophilic AOA.

The current study (as with all genome studies) is limited by existing sequence databases but proposes hypothetical mechanisms for growth of acidophilic archaea that can provide the basis for future experimental testing. Although the presence and activity of previously uncharacterized genes were associated with the acidophilic growth of “*Ca*. Nitrosotalea devanaterra,” one caveat of this approach is that “*Ca*. Nitrosotalea devanaterra” cannot grow at neutral pH. It is therefore impossible to unequivocally confirm that these genes are a prerequisite for acidophily. This question could be further explored by heterologous expression of candidate acidophily genes in a neutrophilic organism (preferably a neutrophilic AOA) to examine whether they confer an acidophilic phenotype on the host. An alternative approach would be site-directed mutagenesis, although unfortunately neither of these techniques has yet been developed for AOA. Further, acetoin production could be determined, transcription of further genes (e.g., Na^+^/solute symporter or Na^+^/H^+^ exchanger genes) studied, and membrane lipid composition determined over a range of pH. Other important future research directions include genome sequencing, lipid profiling, and S-layer glycome profiling of other strains of Nitrosotalea (and potentially acid-tolerant urease-positive AOB).

## Supplementary Material

Supplemental material
