# Supplementary material for: Identifying Potential Mechanisms Enabling Acidophily in the Ammonia-Oxidizing Archaeon “Candidatus Nitrosotalea devanaterra”
Source: Appl Environ Microbiol. 2016 Apr 18;82(9):2608–19. doi: 10.1128/AEM.04031-15 (PMC4836417; doi:10.1128/AEM.04031-15)
Supplement: Supplemental material [file supp_82_9_2608__index.html]

Supplemental material 

# Identifying Potential Mechanisms Enabling Acidophily in the Ammonia-Oxidizing Archaeon “Candidatus Nitrosotalea devanaterra”

## Supplemental material

**Files in this Data Supplement:**

- Supplemental file 1 -

  Similarity of genomic features of "*Ca.* Nitrosotalea devanaterra" and other AOA (Fig. S1); gene organization of one of the unique cell surface modification clusters of "*Ca.* Nitrosotalea devanaterra" (Fig. S2); functional predictions of AmoB (Fig. S3) and AmoC (Fig. S4) of "*Ca.* Nitrosotalea devanaterra"; transcriptional and ammonia oxidation responses of "Ca. Nitrosotalea devanaterra" to pH shock for cell suspensions in inorganic medium (Fig. S5); "*Ca.* Nitrosotalea devanaterra"-specific novel primers used in this study (Table S1); predicted gene annotations of major metabolic pathways in "*Ca.* Nitrosotalea devanaterra" Nd1 (Table S2); phosphate utilization genes in different AOA (Table S3); unique CDs of "*Ca.* Nitrosotalea devanaterra" Nd1 shared by model acidophiles (Table S4); archaeal/V-type ATP synthase subunits of "*Ca.* Nitrosotalea devanaterra" (Table S5); transporters of "*Ca.* Nitrosotalea devanaterra" and other AOA (Table S6); similarity of "*Ca.* Nitrosotalea devanaterra" homeostasis gene cluster (Fig. 2B) to sequences in databases (Table S7); COG- and Pfam-based functional classification of "*Ca.* Nitrosotalea devanaterra" gene clusters involved in cell surface modification (Table S8); characterized ammonium/ammonia transporters in published literature (Table S9); regression analysis of transcript abundance and NO2− production during pH shock (Table S10).

  PDF, 1.3M
