# Supplementary material for: Identifying Potential Mechanisms Enabling Acidophily in the Ammonia-Oxidizing Archaeon “Candidatus Nitrosotalea devanaterra”
Source: Appl Environ Microbiol. 2016 Apr 18;82(9):2608–19. doi: 10.1128/AEM.04031-15 (PMC4836417; doi:10.1128/AEM.04031-15)
Supplement: Supplemental material [file AEM.04031-15_zam999117092so1.pdf]

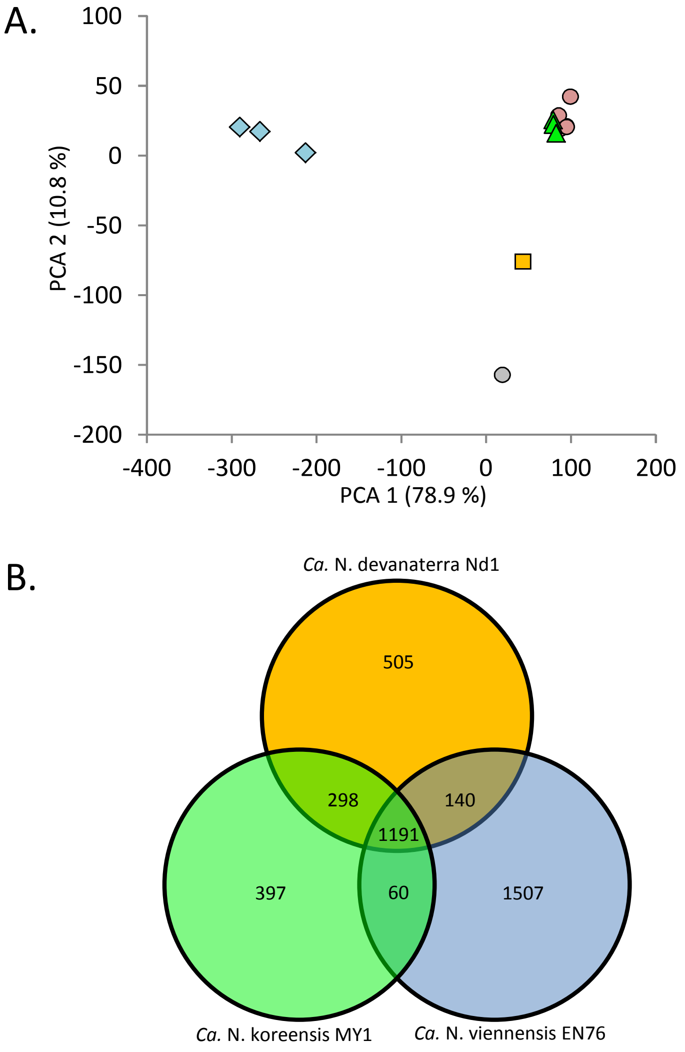

C.

Average nucleotide identity score against *N. devanaterra* Nd1	
N. limnia BG20	68.0
N. limnia SFB1	67.9
N. salaria BD31	67.9
N. maritimus SCM1	67.9
N. koreensis MY1	67.9
Nitrosopumilus sp. SJ	67.8
N. koreensis AR1	67.8
N. sediminis AR2	67.8
N. uzonensis	66.5
N. gargensis	62.9
N. evergladensis	62.7
N. viennensis EN76	62.5

Figure S1: Similarity of genomic features between *'Ca.* N. devanaterra' and other AOA. A. Tetranucleotide word frequency principal component analysis of AOA genomes. Blue diamonds: genus *Nitrososphaera*; green triangles: genus *Nitrosoarchaeum*; pink circles: genus *Nitrosopumilus;* yellow square: '*Ca.* N. devanaterra'; grey circle; '*Ca.* N. uzonensis'. B. Venn diagram of ORFs shared by model terrestrial thaumarchaea. The genome of '*Ca.* N. koreensis MY1' is not closed, and the number of both strain-specific and shared genes may be slightly higher than in the figure. C. Average nucleotide identity (ANI) scores between '*Ca.* N. devanaterra' and other AOA.

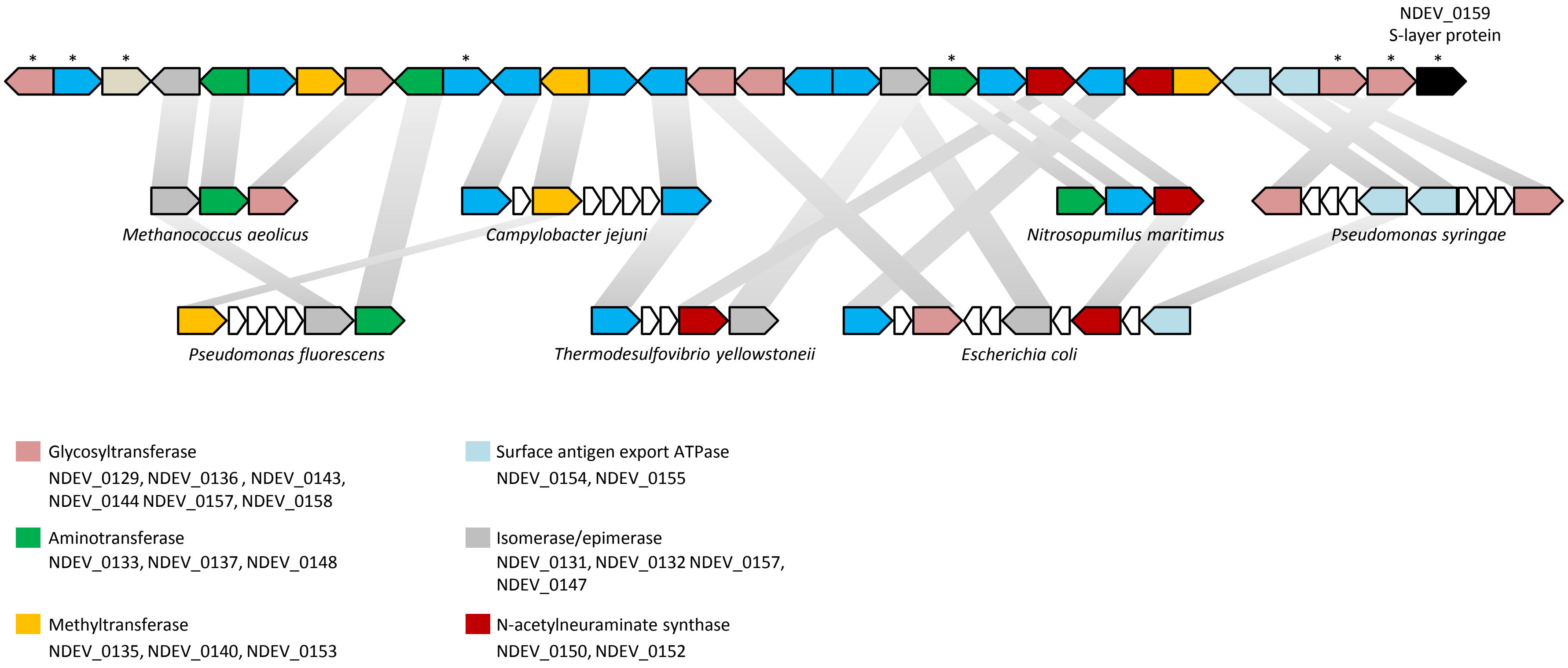

Figure S2: Gene organisation of one of the unique cell surface modification clusters of '*Ca.* N. devanaterra'. Asterisk: greatest amino acid homology to other AOA; no asterisk: greatest similarity to prokaryotes other than AOA. Functional categories of genes are indicated by colours in the inset. Black arrow: S-layer protein; blue arrows: genes with miscellaneous functions (annotations in Tables S1 and S7).

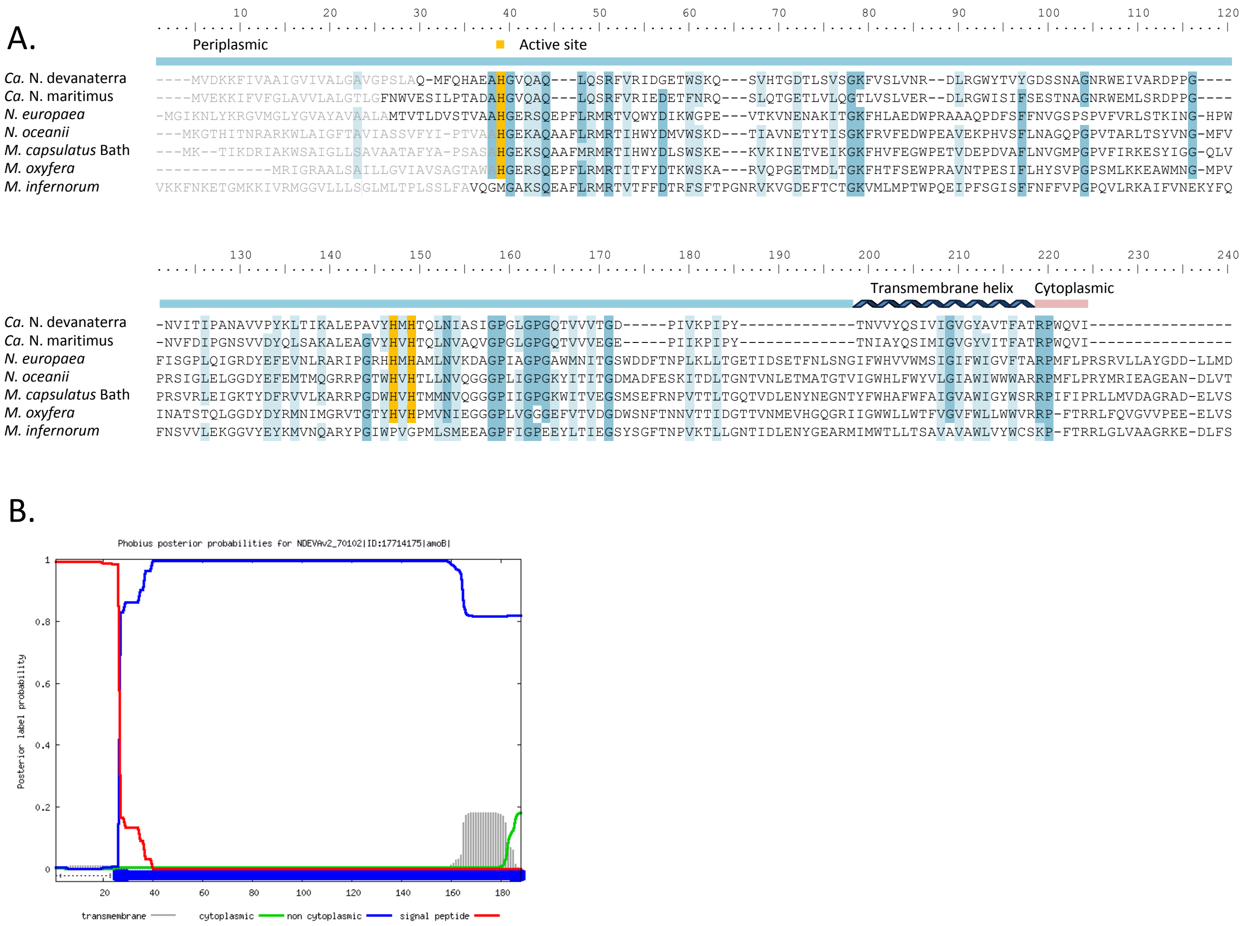

Figure S3: Functional predictions of AmoB of ‘*Ca.* N. devanaterra’. A. Partial amino acid alignment of selected AmoB/PmoB sequences with the predicted active site and protein topology. Residues in grey belong to a signal peptide sequence. Cytoplasmic, periplasmic and transmembrane domains are based on ‘*Ca.* N. devanaterra’ *amoB* sequence, and were similar for all shown sequences. Orange residues denote the active site, blue residues the conservation between different organisms. B. Phobius plot of the transmembrane domains and orientation predictions of AmoB of ‘*Ca.* N. devanaterra’. Probabilities in the Phobius plot are generated by calculating the sum of the total probability that a residue belongs to a helix, cytoplasmic, or non-cytoplasmic summed over all possible paths through a hidden Markov model (1).

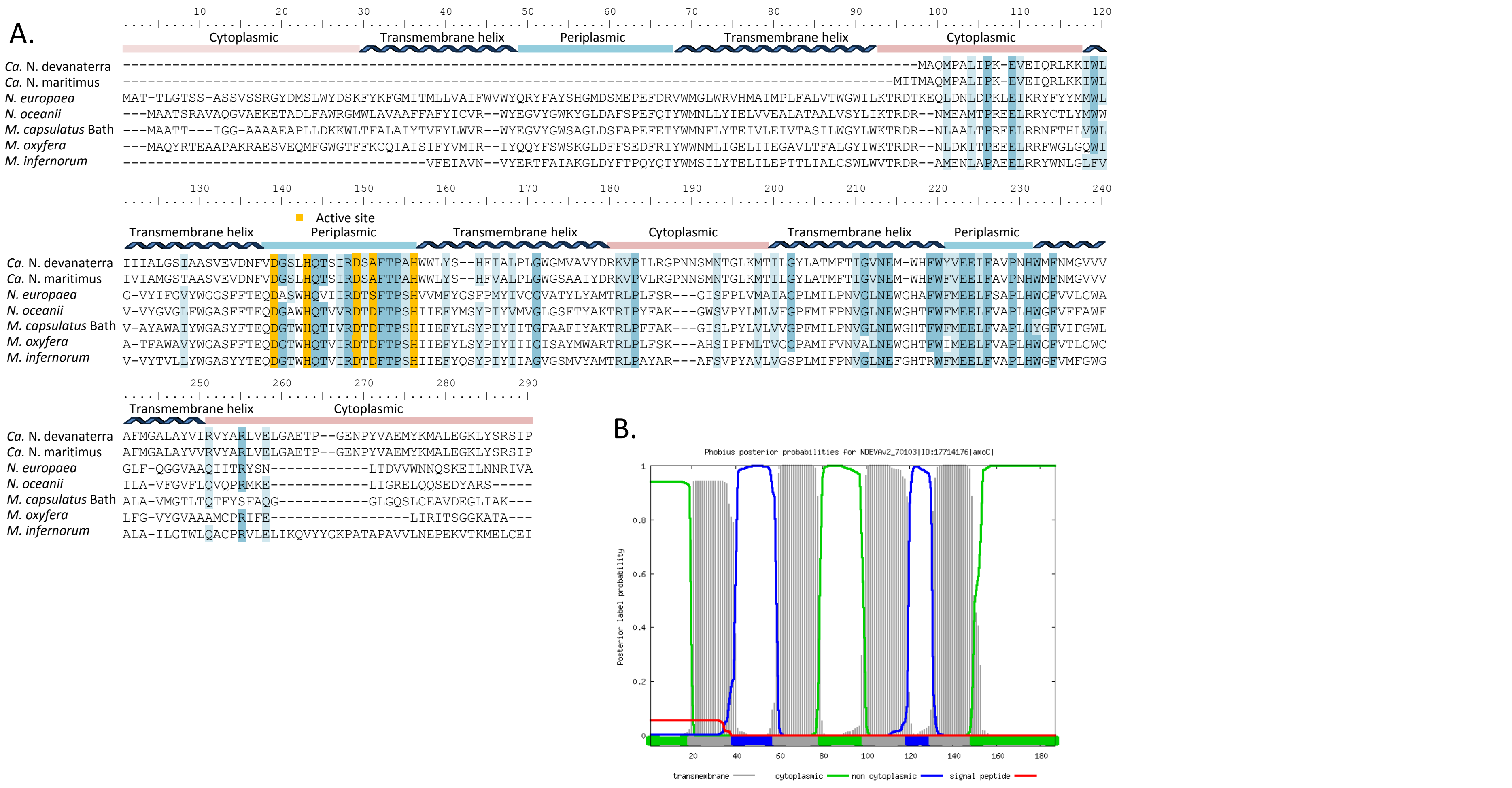

Figure S4: Functional predictions of AmoC of '*Ca.* N. devanaterra'. A. Amino acid alignment of selected AmoC/PmoC sequences with the predicted active site and protein topology. Orange residues denote the active site, blue residues the conservation between different organisms. B. Phobius plot of the transmembrane domains and orientation prediction of AmoC of '*Ca.* N. devanaterra'. Probabilities in the Phobius plot are generated by calculating the sum of the total probability that a residue belongs to a helix, cytoplasmic, or non-cytoplasmic summed over all possible paths through a hidden Markov model (1).

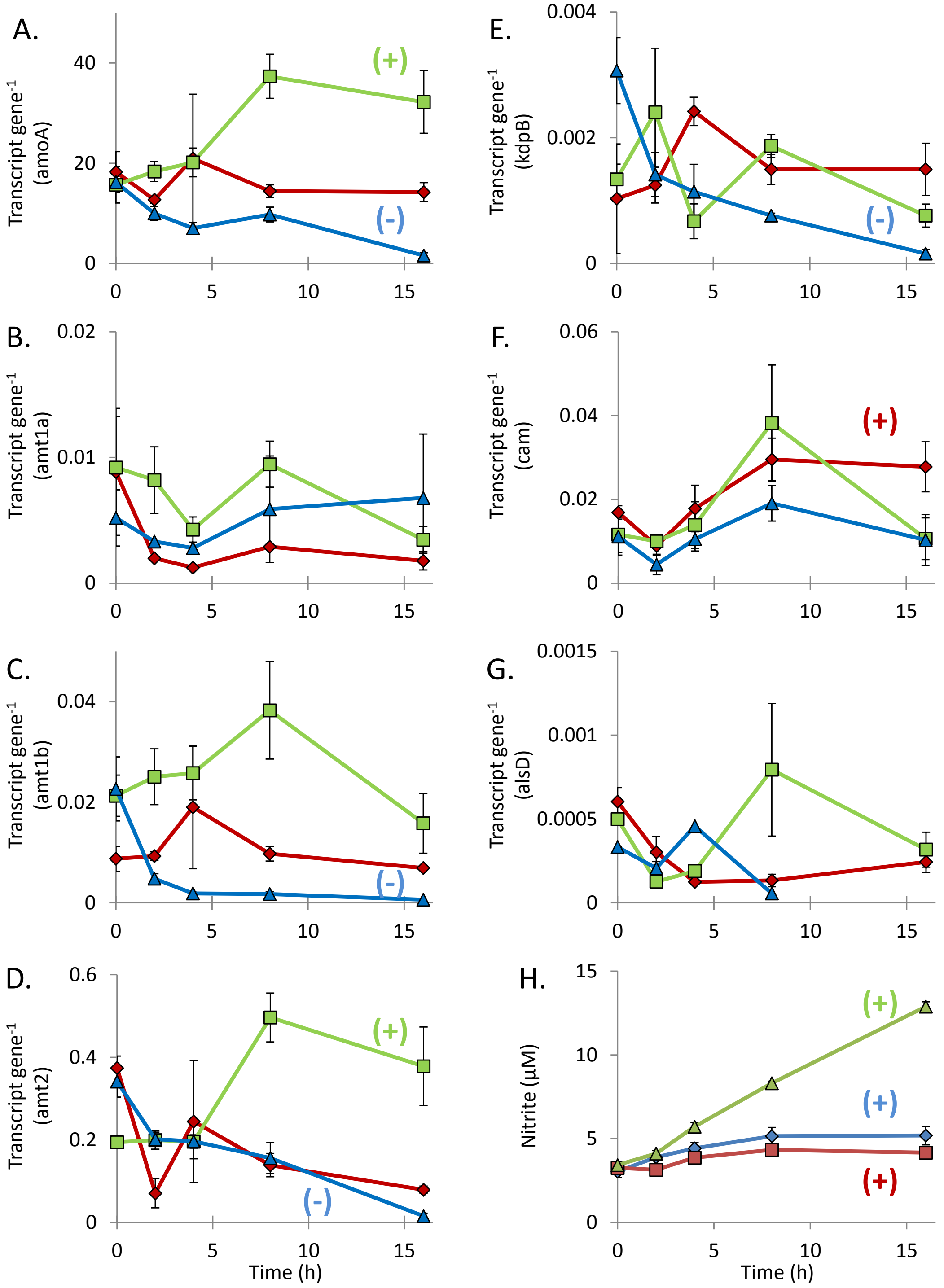

Figure S5: Transcriptional and ammonia oxidation responses of '*Ca.* N. devanaterra' to pH shock in cell suspensions in inorganic medium. A. *amoA* (ammonia mono-oxygenase, subunit A), B. *amt1a* (putative low-affinity ammonium transporter), C. *amt1b* (putative low-affinity ammonium transporter), D. *amt2* (putative high-affinity ammonium transporter), E. *kdpB* (ATPase subunit of potassium transporter), F. cam (carbonic anhydrase), G. *alsD* (acetolactate decarboxylase), H. NO_2^- accumulation during incubation. Colours indicate the pH of each treatment; red: pH 4; green: pH 5; blue: pH 6. + and –indicate a statistically significant (p<0.05) increase or decrease, respectively, in transcription of a specific gene over time as estimated by regression analysis. Abbreviations as in Figure 1, Table S2 and text.

Table S1. *N.devanaterra*-specific novel primers used in this study

Target gene	Locus ID	Gene abbreviation	Primer name	Primer sequence (5′ → 3′)	Amplicon length (bp)	Primer application
Low-affinity ammonium transporter	NDEV_1347	*amt1a*	1F*	CAACATGGTTACAATTCGGG	1649	Standard amplification
			1R*	CCAGCAATACGAGAAAATGG		
			2F	GGAGTAGAGTTTGCTGTTGT	271	qPCR
			2R	CCTCCAAATCCCATCAGTAC		
Low-affinity ammonium transporter	NDEV_1108	*amt1b*	1F	AATTGACACAGGAGACACAG	1255	Standard amplification
			1R	ATCTTGCCAAACTCATCTTCAT		
			2F	TATCACACCCCTATTGTTGAC	207	qPCR
			2R	CGCACTGGTATGTATGACAA		
High-affinity ammonium transporter	NDEV_1784	*amt2*	1F	CTAGCAGATCCTAGCAAGAC	1313	Standard amplification
			1R	CGCCTACTTGAGATATGTCC		
			2F	CATCACTAGCCATTGCTGGTT	235	qPCR
			2R	TCCCAAGAACATTGCGGTAG		
Carbonic anhydrase	NDEV_1082	*Cam*	1F	AATGGCTTCTCCATCCAATG	713	Standard amplification
			1R	CCTCTTCCATCAATGCTTCT		
			2F	TCAAGAGGGCGTAGTGTTAC	205	qPCR
			2R	ACAAGACTCTTCATGCCGAT		
Potassium transporter, ATPase subunit	NDEV_1460	*kdpB*	1F	GCTCAAGGACTCTGTAACAA	2018	Standard amplification
			1R	CAACTCCACCAAATCCGTAT		
			2F	TCTCAGAGTCACAAGCTAGG	227	qPCR
			2R	AGGGTTTGATTCTCCAGTCA		
α-acetolactate decarboxylase	NDEV_1077	*alsD*	1F*	GTTTTGCTTGAAGATGGAGG	1400	Standard amplification
			1R*	CAATGGAAAAGAGGCATACG		
			2F	CAAGCCAGACAAAACATCAG	188	qPCR
			2R	GCAACAACTTCAGATAACGG		

* Primer-binding site is located in the flanking region rather than inside the gene

Supplementary Table 2. Predicted gene annotations of major metabolic pathways in *Ca.* Nitrosotalea devanaterra Nd1

A. Energy generation			
Locus ID	Gene	EC number	Product
Ammonia oxidation			
NDEV_1777	*amoA*	1.14.99.39	Ammonia monooxygenase subunit A
NDEV_1778	*amoX*	_	Uncharacterized protein
NDEV_1779	*amoC*	1.14.99.39	Ammonia monooxygenase subunit C
NDEV_1780	*amoB*	1.14.99.39	Ammonia monooxygenase subunit B
Ammonium transport			
NDEV_0356	*glnB*	_	Nitrogen regulatory protein PII
NDEV_1106	*glnB*	_	Nitrogen regulatory protein P-II
NDEV_1107	*glnB*	_	Nitrogen regulatory protein P-II
NDEV_1108	*amt1b*	_	Ammonium transporter amt1
NDEV_1347	*amt1a*	_	Ammonium transporter amt1
NDEV_1784	*amt2*	_	Ammonium transporter amt2
NDEV_1790	*glnB*	_	Putative nitrogen regulatory protein P-II
Electron transport chain - Complex I			
NDEV_1820	*nuoN*	1.6.99.5	NADH-quinone oxidoreductase subunit N
NDEV_1821	*nuoL*	1.6.99.6	NADH-quinone oxidoreductase subunit L
NDEV_1822	*nuoM*	1.6.99.7	NADH-quinone oxidoreductase subunit M
NDEV_1823	*nuoK*	1.6.99.8	NADH-quinone oxidoreductase subunit K
NDEV_1824	*nuoJ*	1.6.99.9	NADH-quinone oxidoreductase subunit J
NDEV_1825	*nuoI*	1.6.99.10	NADH-ubiquinone oxidoreductase subunit I
NDEV_1826	*nuoH*	1.6.99.11	NADH-quinone oxidoreductase subunit H
NDEV_1827	*nuoD*	1.6.99.12	NADH-quinone oxidoreductase subunit D
NDEV_1828	*nuoC*	1.6.99.13	NADH-ubiquinone oxidoreductase subunit C
NDEV_1829	*nuoB*	1.6.99.14	NADH-quinone oxidoreductase B subunit
NDEV_1830	*nuoA*	1.6.99.15	NADH-ubiquinone oxidoreductase subunit A
Electron transport chain - Complex V / Archaeal ATP synthase			
NDEV_1999	*atpI*	3.6.3.14	Archaeal/V-type ATP synthase subunit I
NDEV_2000	*atpH*	3.6.3.14	Archaeal/V-type ATP synthase subunit H
NDEV_2002	*atpD*	3.6.3.14	Archaeal/V-type ATP synthase subunit D
NDEV_2003	*atpB*	3.6.3.14	Archaeal/V-type ATP synthase subunit B
NDEV_2004	*atpA*	3.6.3.14	Archaeal/V-type ATP synthase subunit A
NDEV_2005	*atpF*	3.6.3.14	Archaeal/V-type ATP synthase subunit F
NDEV_2006	*atpC*	3.6.3.14	Archaeal/V-type ATP synthase subunit C
NDEV_2007	*atpE*	3.6.3.14	Archaeal/V-type ATP synthase subunit E
NDEV_2008	*atpK*	3.6.3.14	Archaeal/V-type ATP synthase subunit K
Electron transport chain - other complexes			
NDEV_0246	*petB*	_	Cytochrome b/b6
NDEV_0161	*coxB*	_	Putative heme-copper oxidase subunit II
NDEV_0162	*coxA*	_	Heme-copper oxidase subunit I
NDEV_0245	*petC*	_	Putative Rieske 2Fe-2S domain cytochrome b6-f complex
NDEV_1844	*sdhB*	1.3.99.1	FeS-center protein of succinate dehydrogenase/fumarate reductase
NDEV_1845	*sdhD*	_	Putative succinate dehydrogenase/fumarate reductase
NDEV_1846	*sdhC*	_	Putative succinate dehydrogenase/fumarate reductase
NDEV_1847	*sdhA*	1.3.99.1	Succinate dehydrogenase, flavoprotein subunit
Electron transport chain - electron carriers			
NDEV_0247	_	_	Copper binding protein, plastocyanin/azurin family
NDEV_0260	_	_	4Fe-4S ferredoxin iron-sulfur binding domain protein
NDEV_0275	_	_	Rieske (2Fe-2S) domain-containing protein
NDEV_0305	_	_	Putative copper-binding protein, plastocyanin/azurin protein
NDEV_0405	_	_	Copper binding protein, plastocyanin/azurin family
NDEV_0408	_	_	Blue (Type1) copper domain-containing protein
NDEV_0413	_	_	4Fe-4S ferredoxin iron-sulfur binding domain protein
NDEV_0450	_	_	Putative Rieske [2Fe-2S] domain protein
NDEV_0609	_	_	Putative blue (Type 1) copper containing protein
NDEV_0610	_	_	Putative blue (Type 1) copper containing protein
NDEV_0615	_	_	Putative blue (Type 1) copper containing protein
NDEV_0780	_	_	Blue (Type1) copper domain-containing protein
NDEV_0814	_	_	Rieske (2Fe-2S) domain protein
NDEV_1075	_	_	Putative secreted copper domain-containing protein

NDEV_1211	_	_	Blue (Type1) copper domain-containing protein
NDEV_1365	_	_	Putative copper-binding protein, plastocyanin/azurin family protein
NDEV_1441	_	_	Blue (Type 1) copper domain protein
NDEV_1530	_	_	Putative Blue (Type 1) copper domain protein
NDEV_1659	_	_	Putative plastocyanin
NDEV_1863	_	_	4Fe-4S ferredoxin iron-sulfur binding domain protein
NDEV_1864	_	_	Putative 4Fe-4S ferredoxin iron-sulfur binding domain protein
NDEV_1992	_	_	Putative Blue (Type 1) copper domain protein
NDEV_2091	_	_	Putative 4Fe-4S ferredoxin iron-sulfur binding domain protein
Electron transport chain - multicopper oxidases			
NDEV_0675	_	_	Putative multicopper oxidase
NDEV_0760	*sir*	1.8.7.1	Putative ferredoxin-dependent sulfite reductase
NDEV_1607	*nirK*	1.7.2.1	Nitrite reductase multicopper oxidase
B. Central carbon metabolism			
Gluconeogenesis			
NDEV_0131	_	5.3.1.9	Putative glucose-6-phosphate/mannose-6-phosphate isomerase
NDEV_0242	*gpmB*	_	Putative 2,3-bisphosphoglycerate-dependent phosphoglycerate mutase
NDEV_0270	*adh*	1.1.1.1	NAD-dependent alcohol dehydrogenase
NDEV_0284	*eno*	4.2.1.11	Enolase
NDEV_0339	*pckA*	4.1.1.49	Phosphoenolpyruvate carboxykinase
NDEV_0454	*pgk*	2.7.2.3	Phosphoglycerate kinase
NDEV_0528	*apmG*	5.4.2.1	2,3-bisphosphoglycerate-independent phosphoglycerate mutase
NDEV_0733	*acsA*	6.2.1.1	Acetyl-coenzyme A synthetase
NDEV_0827	*tpiA*	5.3.1.1	Triosephosphate isomerase
NDEV_1047	*gap*	1.2.1.59	Putative glyceraldehyde 3-phosphate dehydrogenase
NDEV_1055	*fbp*	3.1.3.11	Fructose-1,6-bisphosphatase
NDEV_1169	*fba*	4.1.2.13	Fructose-bisphosphate aldolase
NDEV_1384	*ytsJ*	1.1.1.38	NAD-dependent malic enzyme
NDEV_1848	_	_	Putative isocitrate lyase/PEP phosphonomutase
TCA cycle (reverse and oxidative)			
NDEV_0298	*mdh*	1.1.1.37	Malate dehydrogenase
NDEV_0339	*pckA*	4.1.1.49	Phosphoenolpyruvate carboxykinase
NDEV_0366	*korA*	1.2.7.3	2-oxoglutarate synthase
NDEV_0367	*korB*	1.2.7.3	2-oxoglutarate synthase
NDEV_0829	*sucD*	6.2.1.5	Succinyl-CoA synthetase, NAD(P)-binding, alpha subunit
NDEV_0830	*sucC*	6.2.1.5	Succinyl-CoA ligase [ADP-forming] subunit beta
NDEV_0894	*gltA*	2.3.3.1	Citrate synthase
NDEV_1658	*icd*	1.1.1.41	Isocitrate dehydrogenase (NAD(+))
NDEV_1748	*aco*	4.2.1.3	Aconitate hydratase
NDEV_1844	*sdhB*	1.3.99.1	FeS-center protein of succinate dehydrogenase/fumarate reductase
NDEV_1845	*sdhD*	_	Putative succinate dehydrogenase/fumarate reductase
NDEV_1846	*sdhC*	_	Putative succinate dehydrogenase/fumarate reductase
NDEV_1847	*sdhA*	1.3.99.1	Succinate dehydrogenase, flavoprotein subunit
NDEV_1972	*citE*	4.1.3.6	Citrate lyase
Carbon fixation through 3-hydroxypropionate-4-hydroxybutyrate pathway			
NDEV_0058	*crt*	4.2.1.55	3-hydroxybutyryl-CoA dehydratase
NDEV_1024	_	2.3.1.16	Acetoacetyl-CoA beta-ketothiolase
NDEV_0157	*hbd*	1.1.1.157	3-hydroxybutyryl-CoA dehydrogenase
NDEV_0180	*hbcS*	6.2.1.-	4-hydroxybyturyl-CoA synthetase
NDEV_0181	*hcd*	4.2.1.120, 5.3.3.3	4-hydroxybutyryl-CoA dehydratase/vinylacetyl-CoA-Delta-isomerase
NDEV_0821	_	5.4.99.2	Methylmalonyl-CoA mutase, small subunit
NDEV_0823	*mut*	5.4.99.2	Methylmalonyl-CoA mutase, large subunit
NDEV_0824	_	5.1.99.1	Putative methylmalonyl-CoA epimerase
NDEV_1639	*acsA*	6.2.1.36	3-hydroxypropionyl-CoA synthetase
NDEV_1640	*hpcD*	4.2.1.116	3-hydroxypropionyl-CoA dehydratase
NDEV_1833	*accA*		Biotin carboxyl carrier protein of acetyl-CoA-propionyl-CoA carboxylase
NDEV_1834	*accC*	6.4.1.2, 6.4.1.3	Acetyl-CoA-propionyl-CoA carboxylase, biotin-containing subunit
NDEV_1835	*accB*	6.4.1.2, 6.4.1.3	Acetyl-CoA-propionyl-CoA carboxylase beta subunit
Not found			*Malonyl-CoA reductase*
Not found			*Malonic semialdehyde reductase*

Not found			*Acryloyl-CoA reductase*
Not found			*Succinyl-CoA reductase*
Not found			*Succinic semialdehyde reductase (NADPH)*
Pentose-phosphate pathway			
NDEV_0031	*prs*	2.7.6.1	Ribose-phosphate pyrophosphokinase
NDEV_0131	_	5.3.1.9	Putative glucose-6-phosphate/mannose-6-phosphate isomerase
NDEV_0554	*aroA*	_	2-amino-3,7-dideoxy-D-threo-hept-6-ulosonate synthase
NDEV_1055	*fbp*	3.1.3.11	Fructose-1,6-bisphosphatase
NDEV_1103	_	_	Short-chain dehydrogenase/reductase SDR
NDEV_1169	*fba*	4.1.2.13	Fructose-bisphosphate aldolase
NDEV_1804	*tal*	2.2.1.2	Putative transaldolase
NDEV_1805	*tkt*	2.2.1.1	Putative transketolase C-terminal section
NDEV_1806	*rpe*	2.2.1.1, 5.1.3.1	Ribulose-phosphate 3-epimerase/transketolase
NDEV_1897	*rpiA*	5.3.1.6	Ribose-5-phosphate isomerase A
Unclassified central C metabolism genes			
NDEV_1136	_	_	PfkB family carbohydrate kinase
NDEV_1943	_	_	PfkB family carbohydrate kinase
NDEV_1900	*acyP*	3.6.1.7	Acylphosphatase
NDEV_1309	_	3.1.3.18	Phosphoglycolate phosphatase
C. Cell wall and membrane biosynthesis			
Cell wall synthesis and modification			
NDEV_0129	_	_	Putative glycosyltransferase
NDEV_0130	*cdc*	_	Cell division control protein 6 family protein
NDEV_0131	_	_	Putative glucose-6-phosphate/mannose-6-phosphate isomerase
NDEV_0132	*capD1*	_	Putative UDP-glucose 4-epimerase
NDEV_0133	*spsC1*	_	Pyridoxal phosphate-dependent aminotransferase, cell wall synthesis
NDEV_0134	_	_	Putative Phosphatidylinositol N-acetylglucosaminyltransferase
NDEV_0135	_	_	Putative SAM-dependent methyltransferase
NDEV_0136	_	_	Putative glycosyltransferase
NDEV_0137	*spsC2*	_	Putative pyridoxal phosphate-dependent aminotransferase
NDEV_0138	_	_	GDP-mannose mannosyl hydrolase
NDEV_0139	*gca*	_	GDP-mannose 4,6-dehydratase
NDEV_0140	_	_	Putative methyltransferase
NDEV_0141	_	_	Putative sulfotransferase
NDEV_0142	*cysC*	_	Putative adenylyl-sulfate kinase
NDEV_0143	_	_	Putative glycosyl transferase
NDEV_0144	_	_	Putative glycosyl/glycerophosphate transferase
NDEV_0145	_	_	Putative sialyltransferase
NDEV_0146	_	_	Putative O-acetyltransferase
NDEV_0147	*neuC*	_	UDP-N-acetylglucosamine 2-epimerase
NDEV_0148	*hemL*	_	Glutamate-1-semialdehyde aminotransferase
NDEV_0149	_	_	Oxidoreductase-like protein
NDEV_0150	*neuB1*	_	N-acetylneuraminate synthase
NDEV_0151	*kpsU1*	_	Acylneuraminate cytidylyltransferase
NDEV_0152	*neuB2*	_	Putative N-acetylneuraminate synthase
NDEV_0153	_	_	SAM-dependent methyltransferase
NDEV_0154	*kpsT*	_	Capsular polysaccharide export system, ATPase component
NDEV_0155	*kpsM*	_	Capsular polysaccharide export system, permease component
NDEV_0156	_	_	Protein of unknown function
NDEV_0157	_	_	Putative glycosyltransferase, cell wall synthesis-related
NDEV_0158	_	_	Glycosyltransferase, cell wall synthesis-related
NDEV_0159	*slp1*	_	Putative S-layer protein
NDEV_0197	_	_	Glycosyltransferase, group 1
NDEV_0198	*wecB1*	_	UDP-N-acetylglucosamine 2-epimerase
NDEV_0199	_	_	Putative glycosidase
NDEV_0200	*wecC*	_	UDP-N-acetyl-D-mannosaminuronate dehydrogenase
NDEV_0201	_	_	NAD-dependent epimerase/dehydratase
NDEV_0202	_	_	Putative glycosyltransferase
NDEV_0203	_	_	Conserved protein of unknown function
NDEV_0204	_	_	Short-chain dehydrogenase/reductase SDR
NDEV_0205	*wecB2*	_	UDP-N-acetylglucosamine 2-epimerase
NDEV_0206	_	_	Putative glycosyltransferase

NDEV_0207	_	_	Conserved membrane protein of unknown function
NDEV_0208	*spsC3*	_	Pyridoxal phosphate-dependent aminotransferase, cell wall synthesis
NDEV_0209	_	_	Putative kinase
NDEV_0210	*neuB3*	_	N-acetylneuraminate synthase
NDEV_0211	*kpsU2*	_	Acylneuraminate cytidylyltransferase
NDEV_0212	*neuB4*	_	N-acylneuraminate-9-phosphate synthase
NDEV_0213	_	_	Putative LpxA family acetyltransferase
NDEV_0214	_	_	Conserved protein of unknown function
NDEV_0215	_	_	Putative nucleoside-diphosphate-sugar epimerase
NDEV_0216	_	_	Putative phytanoyl-CoA dioxygenase
NDEV_0217	_	_	Putative uncharacterised deacetylase, LmbE-like
NDEV_0218	_	_	WbqC-like family protein
NDEV_0219	*spsG*	_	Putative pseudaminic acid biosynthesis-associated protein
NDEV_0220	*capD2*	_	UDP-N-acetylglucosamine 4,6-dehydratase (inverting)
NDEV_0221	_	_	Putative glycosyl/glycerophosphate transferase
NDEV_0222	_	_	Putative surface antigen transporter
NDEV_0223	_	_	Putative glycosyltransferase
NDEV_0224	_	_	Putative acyl-CoA N-acyltransferase
NDEV_0225	_	_	Conserved protein of unknown function
NDEV_0226	*spsC4*	_	Putative pyridoxal phosphate-dependent aminotransferase
NDEV_0227	_	_	NAD-dependent epimerase/dehydratase
NDEV_0228	*spsC5*	_	Putative pyridoxal-phosphate dependent aminotransferase
NDEV_0229	_	_	Putative UDP-glucose/GDP-mannose dehydrogenase
NDEV_0230	_	_	NAD-dependent epimerase/dehydratase
NDEV_0294	*slp2*	_	Putative S-layer protein
NDEV_0597	_	6.3.2.4	Putative D-alanine--D-alanine ligase
NDEV_1732	_	_	Phosphoglucomutase/phosphomannomutase subunit alpha/beta
NDEV_1858	_	_	Glycosyl transferase family protein
NDEV_1874	_	_	Phosphoglucomutase/phosphomannomutase, domain II
NDEV_2021	_	_	S-layer domain containing protein
Archaeal membrane biosynthesis			
NDEV_0006	_	2.5.1.41	Geranylgeranylglyceryl phosphate synthase
NDEV_0278	*gds*	2.5.1.10	Geranylgeranyl pyrophosphate synthase
NDEV_0279	*idi*	5.3.3.2	Isopentenyl-diphosphate delta-isomerase
NDEV_0280	_	_	Aspartate/glutamate/uridylate kinase
NDEV_0281	*mvk*	2.7.1.36	Mevalonate kinase
NDEV_0801	_	2.5.1.42	Putative digeranylgeranylglyceryl phosphate synthase
NDEV_1049	*hcs*	2.3.3.10	Hydroxymethylglutaryl-CoA synthase
NDEV_1139	*pgsA*	2.7.8.5	CDP-alcohol phosphatidyltransferase
NDEV_1206	_	_	Geranylgeranyl reductase family protein
NDEV_1923	*mvaA*	1.1.1.34	Hydroxymethylglutaryl-CoA reductase
NDEV_2036	egsA	1.1.1.261	Glycerol-1-phosphate dehydrogenase
Other isoprenoid-related metabolism			
NDEV_0447	*dpm*	2.4.1.83	Dolichyl-phosphate beta-D-mannosyltransferase
NDEV_1351	*uppP*	3.6.1.27	Undecaprenyl-diphosphatase
NDEV_1710	*uppS*	2.5.1.31	Undecaprenyl-diphosphate synthase
NDEV_1819	_	_	Long chain isoprenyl diphosphate synthase
D. Amino acid biosynthesis			
Alanine, aspartate and glutamate metabolism			
NDEV_0011	*glnA*	6.3.1.2	Glutamine synthetase
NDEV_0238	*aspC*	2.6.1.1	Aspartate aminotransferase-like protein
NDEV_0289	*glmS1*	2.6.1.16	Glutamine-fructose-6-phosphate aminotransferase [isomerizing]
NDEV_0397	*glnA*	6.3.1.2	Glutamine synthetase
NDEV_0539	*aspC2*	2.6.1.1	Aspartate aminotransferase-like protein
NDEV_0840	*carB*	6.3.5.5	Carbamoyl-phosphate synthase large subunit
NDEV_0922	_	2.4.2.14	Amidophosphoribosyltransferase
NDEV_1353	*gabD*	1.2.1.79	Putative succinate-semialdehyde dehydrogenase [NADP(+)]
NDEV_1496	*glmS2*	2.6.1.16	Glutamine-fructose-6-phosphate aminotransferase [isomerizing]
NDEV_1608	*ansB*	4.3.1.1	Aspartate ammonia-lyase
NDEV_1621	*argG*	6.3.4.5	Argininosuccinate synthase
NDEV_1643	*gdhA*	1.4.1.3	Glutamate dehydrogenase
NDEV_1714	*purA*	6.3.4.4	Adenylosuccinate synthetase
NDEV_1739	*purB*	4.3.2.2	Adenylosuccinate lyase

NDEV_1842	*argH*	4.3.2.1	Argininosuccinate lyase
NDEV_1903	*gatD*	_	Glutamyl-tRNA(Gln) amidotransferase subunit D
NDEV_1997	*pyrB*	2.1.3.2	Aspartate carbamoyltransferase
Glycine, serine and threonine metabolism pathway			
NDEV_0008	*glyA*	_	Serine hydroxymethyltransferase
NDEV_0059	*thrB*	2.7.1.39	Homoserine kinase
NDEV_0172	_	_	Aspartate/glutamate/uridylate kinase
NDEV_0270	*adh*	1.1.1.1	NAD-dependent alcohol dehydrogenase
NDEV_0364	*gyaR*	1.1.1.26	Glyoxylate reductase
NDEV_0751	_	3.1.3.3	Phosphoserine phosphatase SerB
NDEV_0777	*hom*	1.1.1.3	Homoserine dehydrogenase
NDEV_0889	_	_	Iron-containing alcohol dehydrogenase
NDEV_0891	_	_	Putative MOFRL family protein
NDEV_0953	_	_	8-amino-7-oxononanoate synthase
NDEV_1045	_	4.2.3.1	Threonine synthase
NDEV_1086	*ilvA*	4.3.1.19	Threonine dehydratase
NDEV_1132	*trpB*	4.2.1.20	Tryptophan synthase, beta subunit
NDEV_1133	*trpA*	4.2.1.20	Tryptophan synthase alpha chain
NDEV_1605	_	_	Putative D-isomer specific 2-hydroxyacid dehydrogenase
NDEV_1912	*thrC*	4.2.3.1	Threonine synthase
NDEV_1953	*asd*	1.2.1.11	Aspartate-semialdehyde dehydrogenase
NDEV_2038	_	_	Putative aminotransferase, class V
NDEV_2084	_	2.7.2.4	Putative aspartokinase
Cysteine and methionine metabolism pathway			
NDEV_0019	_	2.5.1.47	Cysteine synthase
NDEV_0115	*metK*	2.5.1.6	Methionine adenosyltransferase 1
NDEV_0172	_	_	Aspartate/glutamate/uridylate kinase
NDEV_0231	*mtnA*	5.3.1.23	Methylthioribose-1-phosphate isomerase
NDEV_0238	*aspC*	2.6.1.1	Aspartate aminotransferase-like protein
NDEV_0298	*mdh*	_	Malate dehydrogenase
NDEV_0411	_	_	Thiosulfate sulfurtransferase
NDEV_0539	_	_	Aminotransferase class I and II
NDEV_0660	*trxB1*	1.8.1.9	Thioredoxin reductase
NDEV_0755	*cysM*	2.5.1.47	Cysteine synthase
NDEV_0756	*trxB2*	1.8.1.9	Thioredoxin reductase
NDEV_0761	_	_	Sulfurtransferase
NDEV_0777	*hom*	1.1.1.3	Homoserine dehydrogenase
NDEV_0815	*ahcY*	3.3.1.1	Adenosylhomocysteinase
NDEV_1613	_	_	Methionine synthase
NDEV_1689	*mtnP*	2.4.2.28	S-methyl-5'-thioadenosine phosphorylase
NDEV_1953	*asd*	1.2.1.11	Aspartate-semialdehyde dehydrogenase
NDEV_2009	_	_	Sulfurtransferase
NDEV_2084	_	2.7.2.4	Putative aspartokinase
Valine, leucine and isoleucine biosynthesis pathway			
NDEV_0170	*ilvE*	2.6.1.42	Putative branched-chain-amino-acid aminotransferase
NDEV_0376	*leuC*	4.2.1.33	3-isopropylmalate dehydratase large subunit 1
NDEV_0377	*leuD*	4.2.1.33	3-isopropylmalate dehydratase small subunit
NDEV_0378	*leuC*	4.2.1.33	3-isopropylmalate isomerase subunit, dehydratase component
NDEV_0379	*leuD*	4.2.1.33	3-isopropylmalate isomerase subunit
NDEV_0820	*ilvC*	1.1.1.86	Ketol-acid reductoisomerase
NDEV_1049	*hcs*	2.3.3.10	Hydroxymethylglutaryl-CoA synthase
NDEV_1126	*ilvD*	4.2.1.9	Dihydroxy-acid dehydratase
NDEV_1171	*ilvB*	2.2.1.6	Putative acetolactate synthase large subunit
NDEV_1172	*ilvH*	2.2.1.6	Putative acetolactate synthase small subunit
NDEV_1173	*leuA*	2.3.3.13	Putative 2-isopropylmalate synthase
NDEV_1174	*leuB*	1.1.1.85	3-isopropylmalate dehydrogenase
NDEV_1853	*ilvE*	2.6.1.42	Branched-chain-amino-acid aminotransferase
Lysine biosynthesis and degradation			
NDEV_0172	_	_	Aspartate/glutamate/uridylate kinase
NDEV_0377	*leuD*	_	3-isopropylmalate dehydratase small subunit
NDEV_0777	*hom*	1.1.1.3	Homoserine dehydrogenase
NDEV_1628	*aksA*	2.3.3.14	Putative homocitrate synthase AksA
NDEV_1631	_	_	N-acetyl-ornithine/N-acetyl-lysine deacetylase

NDEV_1953	*asd*	1.2.1.11	Aspartate-semialdehyde dehydrogenase	
NDEV_2084	_	2.7.2.4	Putative aspartokinase	
NDEV_0058	*crt*	4.2.1.55	3-hydroxybutyryl-CoA dehydratase	
NDEV_0396	_	_	Lysine 2,3-aminomutase related protein	
NDEV_1640	*hpcD*	_	3-hydroxypropionyl-CoA dehydratase	
Arginine and proline metabolism				
NDEV_0011	*glnA*	6.3.1.2	Glutamine synthetase	
NDEV_0238	*aspC*	2.6.1.1	Aspartate aminotransferase-like protein	
NDEV_0344	*argF*	2.1.3.3	Ornithine carbamoyltransferase	
NDEV_0397	*glnA*	6.3.1.2	Glutamine synthetase	
NDEV_0476	*argD2*	2.6.1.11	Putative Acetylornithine aminotransferase	
NDEV_0803	*speB*	3.5.3.11	Agmatinase	
NDEV_0838	*carA*	6.3.5.5	Carbamoyl-phosphate synthase small chain	
NDEV_0840	*carB*	6.3.5.5	Carbamoyl-phosphate synthase large subunit	
NDEV_1070	_	_	Putative agmatinase	
NDEV_1110	*gatA*	_	Glutamyl-tRNA(Gln) amidotransferase subunit A	
NDEV_1165	*pdaD*	4.1.1.19	Pyruvoyl-dependent arginine decarboxylase	
NDEV_1223	*putB*	1.5.99.8	Proline dehydrogenase	
NDEV_1224	*putC*	1.5.1.12	1-pyrroline-5-carboxylate dehydrogenase	
NDEV_1621	*argG*	6.3.4.5	Argininosuccinate synthase	
NDEV_1624	*argC*	1.2.1.38	N-acetyl-gamma-glutamyl-phosphate/N-acetyl-gamma-aminoadipyl-phosphate reductase	
NDEV_1625	*argB*	2.7.2.8	Acetylglutamate/acetylaminoadipate kinase	
NDEV_1626	*argD*	2.6.1.11	Acetylornithine aminotransferase	
NDEV_1643	*gdhA*	1.4.1.3	Glutamate dehydrogenase	
NDEV_1842	*argH*	4.3.2.1	Argininosuccinate lyase	
Histidine metabolism				
NDEV_1914	*hisI*	3.5.4.19	Phosphoribosyl-AMP cyclohydrolase	
NDEV_1915	*hisF*	_	Imidazole glycerol phosphate synthase, catalytic subunit with HisH	
NDEV_1916	*hisA*	5.3.1.16	1-(5-phosphoribosyl)-5-[(5-phosphoribosylamino)methylideneamino] imidazole-4-carboxamide isomerase	
NDEV_1918	*hisB*	4.2.1.19	Imidazoleglycerol-phosphate dehydratase	
NDEV_1920	*hisC*	2.6.1.9	Histidinol-phosphate aminotransferase	
NDEV_1921	*hisD*	1.1.1.23	Histidinol dehydrogenase	
NDEV_1922	*hisG*	2.4.2.17	ATP phosphoribosyltransferase	
Phenylalanine, tryptophan and tyrosine biosynthesis				
NDEV_0238	*aspC*	2.6.1.1	Aspartate aminotransferase-like protein	
NDEV_0538	*tyrA*	1.3.1.12	Prephenate dehydrogenase	
NDEV_0540	*aroC*	4.2.3.5	Chorismate synthase	
NDEV_0541	*aroA*	2.5.1.19	Putative 3-phosphoshikimate 1-carboxyvinyltransferase	
NDEV_0542	*aroK*	2.7.1.71	Shikimate kinase	
NDEV_0543	*aroE*	1.1.1.25	Shikimate dehydrogenase	
NDEV_0547	*aroD*	4.2.1.10	3-dehydroquinate dehydratase	
NDEV_0553	*aroB*	1.4.1.24	3-dehydroquinate synthase	
NDEV_0554	*aroA*	2.2.1.10	2-amino-3,7-dideoxy-D-threo-hept-6-ulosonate synthase	
NDEV_1128	*trpE*	4.1.3.27	Anthranilate synthase component 1	
NDEV_1129	*trpG*	4.1.3.27	Fragment of Anthranilate synthase	
NDEV_1130	*trpD*	2.4.2.18	Anthranilate phosphoribosyltransferase	
NDEV_1131	*trpC*	4.1.1.48	Indole-3-glycerol-phosphate synthase	
NDEV_1132	*trpB*	4.2.1.20	Tryptophan synthase, beta subunit	
NDEV_1133	*trpA*	4.2.1.20	Tryptophan synthase alpha chain	
NDEV_1920	*hisC*	2.6.1.9	Histidinol-phosphate aminotransferase	
NDEV_1935	*pheA*	4.2.1.51	Prephenate dehydratase	
E. Putative pH homeostasis gene clusters (in addition to homologues in Supplementary Table 3)				
Kdp potassium transporter gene cluster				Figure 2A
NDEV_1454	_	_	Putative PAS/PAC sensor histidine kinase	
NDEV_1455	*kdpD*	2.7.13.3	K+ channel signal transduction histidine kinase	
NDEV_1456	_	_	Peptidase M10A and M12B matrixin and adamalysin	3
NDEV_1457	_	_	K+ channel signal transduction response regulator	2
NDEV_1458	_	_	Protein of unknown function	1
NDEV_1459	*kdpC*	3.6.3.12	Potassium-transporting ATPase C chain	
NDEV_1460	*kdpB*	3.6.3.12	Potassium-transporting ATPase B chain	
NDEV_1461	*kdpA*	3.6.3.12	Potassium-transporting ATPase A chain	

pH homeostasis genomic island				Figure 2B
NDEV_1073	_	_	Putative transposase	9
NDEV_1074	_	_	Putative adenylate cyclase	8
NDEV_1075	_	_	Putative secreted copper domain-containing protein	7
NDEV_1076	_	_	Response regulator receiver protein	6
NDEV_1077	*alsD*	4.1.1.5	Putative alpha-acetolactate decarboxylase	
NDEV_1078	_	_	Protein of unknown function	5
NDEV_1079	*mgtA*	3.6.3.2	Magnesium-transporting P-type ATPase	
NDEV_1080	*uspA*	_	UspA domain-containing protein	
NDEV_1081	_	4.6.1.1	Adenylate/guanylate cyclase sensor protein	4
NDEV_1082	*cam*	4.2.1.1	Carbonic anhydrase	
NDEV_tRNA27	_	_	Val tRNA	3
NDEV_1083	_	_	Conserved protein of unknown function	2
NDEV_1084	_	_	Phosphoribosyltransferase-like protein	1
NDEV_1085	*mntH*	_	NRAMP family Mn2+/Fe2+ transporter	
F. Protection from stress				
Protection from oxidative stress and protein folding				
NDEV_0113	_	_	DnaJ class molecular chaperone	
NDEV_0185	*trxA1*	_	Thioredoxin	
NDEV_0341	*sodA*	1.15.1.1	Superoxide dismutase	
NDEV_0478	*ygaF*	1.11.1.15	Putative peroxiredoxin	
NDEV_0522	_	_	Molecular chaperone	
NDEV_0529	_	5.2.1.8	FKBP-type peptidyl-prolyl cis-trans isomerase	
NDEV_0559	*ygaF*	1.11.1.15	Putative peroxiredoxin YgaF	
NDEV_0657	*dps*	1.16.-.-	DNA protection during starvation protein	
NDEV_0660	*trxB1*	1.8.1.9	Thioredoxin reductase	
NDEV_0756	*trxB2*	1.8.1.10	Thioredoxin reductase	
NDEV_0951	_	_	Redoxin domain protein	
NDEV_1048	_	_	DSBA-like thioredoxin domain protein	
NDEV_1122	_	_	Thioredoxin	
NDEV_1166	*bcp*	1.11.1.15	Putative peroxiredoxin	
NDEV_1327	_	_	Putative Copper resistance family protein	
NDEV_1598	_	_	Alkyl hydroperoxide reductase/ Thiol specific antioxidant/ Mal allergen	
NDEV_1769	_	_	Alkyl hydroperoxide reductase/ Thiol specific antioxidant/ Mal allergen	
NDEV_1832	*bcp*	1.11.1.15	Putative peroxiredoxin	
NDEV_1868	*trxA2*	_	Thioredoxin	
NDEV_1906	*dnaJ*	_	Chaperone Hsp40, co-chaperone with DnaK	
NDEV_1907	*dnaK*	_	Chaperone protein DnaK	
NDEV_2098	_	_	Molecular chaperone (Small heat shock protein)	
G. Motility				
Flagellar motility and chemotaxis				
NDEV_0982	*flaJ*	_	Archaeal flagella assembly protein J	
NDEV_0983	*flaI*	_	Putative archaeal flagellar protein I homolog	
NDEV_0984	*flaH*	_	Flagellar accessory protein FlaH	
NDEV_0985	*flaF*	_	Putative flagella protein	
NDEV_0986	*flaG*	_	Flagellar protein FlaG	
NDEV_0988	*flaB1*	_	Archaeal flagellin	
NDEV_0990	*flaB2*	_	Archaeal flagellin	
NDEV_0991	*flaB3*	_	Archaeal flagellin	
NDEV_0992	*flaB4*	_	Archaeal flagellin	
NDEV_0993	_	_	Putative methyl-accepting chemotaxis protein	
NDEV_0996	*cheW*	_	Chemotaxis protein CheW	
NDEV_0997	*cheY*	_	Chemotaxis response regulator CheY	
NDEV_0998	*cheB*	3.1.1.61	Chemotaxis response regulator protein-glutamate methylesterase	
NDEV_0999	*cheA*	2.7.13.3	Chemotaxis histidine kinase CheA	
NDEV_1000	*cheC*	_	Putative chemotaxis protein CheC	
NDEV_1001	*cheR*	_	CheR chemotaxis protein methyltransferase	
NDEV_1002	*ccdB*	_	Protein CcdB	
NDEV_1003	*cheC*	_	Putative chemotaxis protein CheC	
NDEV_1004	*cheD*	3.5.1.44	Putative chemoreceptor glutamine deamidase CheD	
NDEV_1009	*CheY*	_	Putative chemotaxis response regulator CheY	
NDEV_1011	_	_	CheY-like receiver	
NDEV_1012	_	_	CheY-like receiver	

NDEV_1015	_	_	CheY-like receiver	
NDEV_1020	*flaK*	_	Putative archaeal preflagellin peptidase	
Additional putative chemotaxis / signal receptors				
NDEV_0249	_	_	CheY-like receiver	
NDEV_0433	_	_	CheY-like receiver	
NDEV_0649	_	_	CheY-like receiver	
NDEV_0739	_	_	CheY-like receiver	
NDEV_0981	_	_	CheY-like receiver	
NDEV_1076	_	_	CheY-like receiver	
NDEV_1395	_	_	CheY-like receiver	
NDEV_1422	_	_	CheY-like receiver	
Gas vacuole formation				
NDEV_1498	*gvpD*	_	Putative gas vesicle regulatory protein GvpD	
NDEV_1502	*gvpA*	_	Putative gas vesicle structural protein GvpA	
NDEV_1504	*gvpJ*	_	Putative gas vesicle structural protein GvpJ	
NDEV_1505	*gvpK*	_	Putative gas vesicle protein GvpK	
NDEV_1506	*gvpL*	_	Putative gas vesicle synthesis protein GvpL_GvpF	
NDEV_1507	*gvpG*	_	Putative gas vesicle protein GvpG	
H. Phosphorus utilisation				
Phosphate uptake and utilisation				Figure S6
NDEV_0495	*sixA*	3.1.3.-	Phosphohistidine phosphatase	
NDEV_0496	*ppx*	3.6.1.11	Putative Exopolyphosphatase	
NDEV_0497	_	_	Conserved protein of unknown function	6
NDEV_0498	*pit*	_	Putative phosphate permease	
NDEV_0499	_	_	Putative pit accessory protein	1
NDEV_0500	*phoU1*	_	Phosphate uptake regulator	
NDEV_0501	*pstC*	3.6.3.-	Phosphate ABC transporter, permease component	
NDEV_0502	*pstA*	3.6.3.-	Phosphate ABC transporter, permease component	
NDEV_0503	*pstB*	3.6.3.-	Phosphate ABC transporter, ATPase component	
NDEV_0504	*phoU2*	_	Phosphate uptake regulator	
NDEV_0505	*pstS1*	3.6.3.-	Phosphate ABC transporter, periplasmic component	
NDEV_0506	*pstS2*	3.6.3.-	Phosphate ABC transporter, periplasmic component	
NDEV_1167	*ppa*	3.6.1.1	Inorganic pyrophosphatase	
NDEV_1962	_	_	Phosphate uptake regulator, PhoU	
NDEV_2026	_	_	Phosphate uptake regulator, PhoU	
I. Hydrogenase / formate hydrogen lyase				
NDEV_0880	*hyfB*	1.6.99.5	NiFe-hydrogenase-4 subunit B	
NDEV_0881	*hyfC*	1.6.99.6	NiFe-hydrogenase-4, membrane subunit	
NDEV_0882	*hyfE*	1.6.99.7	NiFe-hydrogenase-4, subunit E	
NDEV_0883	*hyfF*	1.6.99.8	NiFe-hydrogenase-4, membrane subunit	
NDEV_0884	*hyfG*	1.6.99.9	NiFe-hydrogenase-4, large subunit	
NDEV_0885	*hyfI*	1.6.99.10	NiFe-hydrogenase/NADH-quinone oxidoreductase	

Supplementary Table 3. Phosphate utilisation genes in different AOA

Organism	Low-affinity uptake(pit)		High-affinity uptake (pst)					Putative phosphonate ABC transporter			Phosphonate biosynthesis				Poly-P biosynthesis and utilisation	
	pit	pit accessory protein	pstA	pstB	pstC	pstS	phoU				mpnS	ppd	ppm	pdh	ppk	ppx
Ca. N. devanaterra Nd1	1	1	1	1	1	2	2	-	-	-	-	-	-	-	P	1
Ca. N. maritimus SCM1	1	1	1	1	1	1	2	1	1	1	1	1	1	1	P	-
Ca. Nitrosopumilus sp. SJ	1	1	-	-	-	-	1	-	-	-	-	-	-	-	P	-
Ca. N. koreensis AR1	1	1	-	-	-	-	1	-	-	-	-	-	-	-	P	-
Ca. N. sediminis AR2	1	1	-	-	-	-	-	1	1	1	-	-	-	-	P	-
Ca. N. salaria BD31	-	-	-	-	-	-	-	-	-	-	P*	-	-	P*	P	-
Ca. N. limnia SFB1	1	1	1	1	1	1	2	-	-	-	-	1	1	1	P	-
Ca. N. limnia BG20	1	1	-	-	-	-	1	-	-	-	-	-	-	-	P	-
Ca. N. koreensis MY1	1	1	1	1	1	1	2	-	-	-	-	-	-	-	P	-
Ca. N. uzonensis N4	1	1	1	1	1	1	2	1	1	1	-	-	-	-	P	1
Ca. C. symbiosum	-	-	1	1	1	1	2	1	1	1	-	-	-	-	P	-
Ca. N. gargensis	1	1	1	1	1	3	3	-	-	-	-	-	-	-	P	-

Numerals indicate the numbers of homologues per genome. P=putative, *AE1Lv1_170001 and AE1Lv1_350001 are on very short contigs without any flanking ORFs, synteny could not be confirmed. Similarity to *N. maritimus* SCM1 homologues is high (70.96% and 77.47%, respectively).

Supplementary Table 4. Unique CDs of *Ca.* N. devanaterra Nd1 shared by model acidophiles

Locus ID	Product	Archaea							Bacteria						AOA		
		Metallosphaera sedule	*Picrophilus torridus*	*Sulfolobus acidocaldarius*	*Sulfolobus solfataricus*	*Sulfolobus tokodaii*	*Thermoplasma acidophilum*	*Thermoplasma volcanium*	*Acidophilum multivorum*	*Acidithiobacillus caldus*	*Acidithiobacillus ferrivorans*	*Acidithiobacillus ferrooxidans*	*Catenulispora acidiphila*	*Methylacidiphilum infernorum*	*Ca.* N. viennensis	*Ca.* N. evergladensis	All other AOA
NDEV_0016	GCN5-related N-acetyltransferase									1							
NDEV_0236	Uncharacterised domain UPF0150 protein				1												
NDEV_0252	Protein of unknown function												1				
NDEV_0373	Protein of unknown function DUF347								1	1	1	1	1				
NDEV_0529	FKBP-type peptidyl-prolyl cis-trans isomerase	1		1	1	1				1	1	1					
NDEV_0552	Protein of unknown function			1													
NDEV_0570	Putative transcriptional regulator, CopG/Arc/MetJ family	1			1												
NDEV_0578	Putative Type-1 restriction enzyme MjaXIP specificity protein							1									
NDEV_0651	Coiled-coil motif protein				1												
NDEV_0721	Protein of unknown function													1			
NDEV_0724	Protein of unknown function	1				1											
NDEV_0771	Exported protein of unknown function										1	1					
NDEV_0843	Formylmethanofuran dehydrogenase subunit E region						1	1									
NDEV_0844	Putative permease						1	1									
NDEV_1032	Putative AAA ATPase			2													
NDEV_1078	Protein of unknown function								1								
NDEV_1079	Magnesium-transporting P-type ATPase								1			2		1			
NDEV_1085	NRAMP family Mn2+/Fe2+ transporter								1					1			
NDEV_1113	Putative dioxygenase												1				
NDEV_1157	Exported protein of unknown function					1											
NDEV_1159	Exported protein of unknown function				1												
NDEV_1231	Major facilitator superfamily transporter	4	4	5	4	4	4	3					8				
NDEV_1266	Conserved protein of unknown function			1													
NDEV_1276	Membrane protein of unknown function				1												
NDEV_1296	Putative ATP-dependent DNA ligase												1	1			
NDEV_1297	Na+/solute symporter	1	1	2	3	2	1	1	1	1			1				
NDEV_1333	Exported protein of unknown function				1												
NDEV_1368	Chromosome segregation ATPase-like protein													1			
NDEV_1416	Protein of unknown function					1											
NDEV_1448	Major facilitator superfamily transporter	1	1		1				2								
NDEV_1462	Putative transcriptional regulator, AsnC family	1			1												
NDEV_1486	Putative transcriptional regulator			1													
NDEV_1509	Spherulin-4-like protein						1										
NDEV_1562	Protein of unknown function				1												
NDEV_1577	Membrane protein of unknown function							1									
NDEV_1669	Exported protein of unknown function										1	1					
NDEV_1703	Protein of unknown function					1											
NDEV_1882	Putative AAA ATPase	1															
NDEV_1883	Protein of unknown function						1										
NDEV_1999	Archaeal/V-type ATP synthase subunit I		1				1	1									
NDEV_2002	Archaeal/V-type ATP synthase subunit D		1				1	1									
NDEV_2005	Archaeal/V-type ATP synthase subunit F		1				1	1									
NDEV_2006	Archaeal/V-type ATP synthase subunit C		1				1	1									
NDEV_2044	Putative phage protein					1											
NDEV_2054	Conserved protein of unknown function													1			
NDEV_1077	α-acetolactate decarboxylase																
NDEV_1082	Carbonic anhydrase	1				1	1	1							1	1	
NDEV_1443	NRAMP family Mn2+/Fe2+ transporter								1							1	
NDEV_1447	Na+/H+ exchanger																
NDEV_1460	Potassium-transporting ATPase B chain		1				1	1	1	2	1	2	1	1	1	1	
NDEV_1461	Potassium-transporting ATPase A chain		1				1	1	1	3	1	3	1	1	1	2	
NDEV_1587	Na+/H+ exchanger																

Blue indicates conservation between '*Ca.* N. devanaterra' and model acidophiles, and grey between '*Ca.* N. devanaterra' and Thaumarchaeota. Numerals indicate the number of homologues per genome.

Supplementary Table 5. Archaeal / V-type ATP synthase subunits of *Ca.* N. devanaterra

Locus ID	Gene	Subunit	Domain	Best non-AOA hit			Best uncultured hit			Best cultivated AOA hit		
				Organism	%	e-value	Marine fosmid	%	e-value	Organism	%	e-value
NDEV_1999	*atpI*	*a*	A_0	*Thermoplasma volcanium*	38.22	8e-157	HF4000_ANIW137N13	58.16	0	*Ca.* N. koreensis AR1	24.33	3e-26
NDEV_2008	*atpK*	*c*	A_0	*Micrarchaeum acidiphilum*	91.3	1e-34	HF4000_ANIW97J3	76.64	2e-44	Ca. N. koreensis AR1	38.89	3e-9
NDEV_2006	*atpC*	C	Central stalk	*Picrophilus torridus*	34.96	9e-65	HF4000_ANIW97J3	68.27	3e-169	*Ca.* N. gargensis	25.84	1e-19
NDEV_2002	*atpD*	D	Central stalk	*Thermoplasma acidophilum*	44.33	5e-51	HF4000_APKG10D8	73.87	3e-106	*Ca.* N. gargensis	29.05	2e-23
NDEV_2005	*atpF*	F	Central stalk	*Micrarchaeum acidiphilum*	44.44	1e-18	HF4000_APKG10D8	57.52	1e-30	*Ca.* N. uzonensis N4	23.00	1e-3
NDEV_2007	*atpE*	E	Peripheral stalk	*Archeoglobus profundus*	27.42	2-13	HF4000_ANIW97J3	51.63	5e-63	*Ca.* N. uzonensis N4	22.11	4e-10
NDEV_2000	*atpH*	H	Peripheral stalk	*Methanocaldococcus fervens*	33.01	2e-7	HF4000_ANIW137N13	50.94	4e-29	*Ca.* N. viennensis EN76	28.18	1e-05
NDEV_2004	*atpA*	A	A_1	*Ferroplasma acidarmanus*	63.79	0	HF4000_APKG9M20	82.59	0	*Ca.* N. uzonensis N4	54.53	0
NDEV_2003	*atpB*	B	A_1	*Thermoplasma acidophilum*	71.15	0	HF4000_ANIW137N13	83.95	0	*Ca.* N. koreensis AR1	59.29	0

Supplementary Table 6. Transporters of *Ca.* N. devanaterra and other AOA

	***Ca.* N. devanaterra**	*Ca.* N. maritimus SCM1	*Ca.* N. koreensis AR1	*Ca.* N. sediminis AR2	*Ca.* N. koreensis MY1	*Ca.* N. gargensis Ga9.2	*Ca.* N. viennensis EN76
Primary transporters							
ABC	**36**	35	21	25	28	44	48
ArsAB	**1**				1	1	3
DNA-T						1	
F-ATPase	**10**	9	9	8	8	11	10
H+-Ppase		1	1	1	1	1	
P-ATPase	**4**			1		1	5
IVSP				1			
Secondary transporters							
AAAP			1				
APC	**2**	1	1	1	1	3	2
ArsB					1	1	2
BASS						2	
CaCA	**1**	1	1	1		2	1
CDF	**1**	3	1	1	2	4	4
CPA1	**2**						
CPA2		3	5	5	5	9	5
DAACS		1	1	2			
DASS	**2**			1			
DMT	**3**	3		1	2	2	1
FNT						1	
GPH						1	1
LCT						1	
MFS	**9**	3	4	5	8	11	8
MOP		1			1	1	1
NCS						1	3
NiCoT	**1**	1		1	1	1	
Nramp	**2**	1	1	1	2		
PiT	**1**		1	1	1	1	1
RhtB		1	1	2	1	1	1
RND							1
SBT	**2**	1	1	1			
SSS	**1**	4	2	3		3	7
SulP		1					
Tat	**3**	3	3	3	3	3	3
Trk		1	1	2	2	4	
VIT	**1**		1	1	1	1	1
ZIP	**1**	2	1	1	1	1	2
Other transporters							
FeoB	**1**	1	1	1		1	1
HCC	**1**	2	1	1	1	1	
ILT		1	1	1	1	1	1
MerTP						1	
MgtE	**1**	1	1		1	2	4
TerC					1	2	1
UBS1						1	
LIVCS							1
UT							1
Ion channels							
Amt	**3**	2	2	2	2	3	3
LIC						2	2
MIP	**2**	2	2	2	2	2	1
MIT	**1**	2	2	2	1	2	2
MscS	**2**	5	5	4	2	4	3
MscL	**1**		1		1	1	1
TRIC	**1**	1	1	1	1	1	1
UT						1	
VIC	**1**	1	1	1		1	

Numerals indicate the number of homologues per genome.

Supplementary Table 7. Similarity of *Ca.* N devanaterra homeostasis gene cluster (Figure 2B) to sequences in databases

Locus ID	Product	AOA			Other organisms		
		Organism	%	e-value	Organism	%	e-value
NDEV_1073	Putative transposase	No hit			*Mycoplasma agalactiae*	45	0.003
NDEV_1074	Putative adenylate cyclase	No hit			No hit		
NDEV_1075	Putative secreted copper domain-containing protein	*Ca.* Nitrosopumilus sp. AR2	30.22	3e-13	*Paracoccus versutus*	43.59	0.002
NDEV_1076	CheY-like receiver	*Ca.* N. uzonensis N4	45.9	2e-27	*Azotobacter vinelandii*	40.51	8e-9
NDEV_1077	Putative alpha-acetolactate decarboxylase	No hit			*Calothrix* sp. PCC7507	40.61	8e-49
NDEV_1078	Protein of unknown function	No hit			No hit		
NDEV_1079	Magnesium-transporting P-type ATPase	No hit			*Methanomethylovorans hollandica*	61.54	0
NDEV_1080	UspA domain-containing protein	*Ca.* Nitrosopumilus sp. AR2	43.26	5e-29	*Halomonas elongata*	29.86	1e-8
NDEV_1081	Adenylate/guanylate cyclase sensor protein	*Ca.* N. uzonensis N4	48.08	9e-89	*Bacillus cereus*	30.09	1e-6
NDEV_1082	Carbonic anhydrase	*Ca.* N. viennensis EN76	54.04	1e-106	*Methanosalsum zhilinae*	46.23	1e-48
NDEV_1083	Conserved protein of unknown function	*Ca.* Nitrosopumilus sp. AR2	32.03	3e-24	No hit		
NDEV_1084	Phosphoribosyltransferase-like protein	*Ca.* N. maritimus SCM1	60.94	3e-49	*Caldisphaera lagunensis*	33.96	1e-14
NDEV_1085	NRAMP family Mn2+/Fe2+ transporter	No hit			*Ktedonobacter racemifer*	44.22	2e-109

Table S8: COG and Pfam based functional classification of *Ca*. N. devanaterra gene clusters involved in cell surface modification. AOA column indicates whether a homologue is present (Y) or absent (N) in other AOA. 37 out of 65 CDS were associated with the COG class 'M' for 'Cell wall/membrane/envelop biogenesis'. Other COG classes: E: Amino Acid metabolis and transport; F: Nucleotide metabolism and transport; G: Carbohydrate metabolism and transport; H: Coenzyme metabolism; I: Lipid metabolism; L: Replication and repair; O: Post-translational modification, protein turnover, chaperone functions; P: Inorganic ion transport and metabolism; Q: Secondary Structure; R: General Functional Prediction only; S: Function Unknown.

Supplementary Table 8. Gene clusters of Ca. N. devanaterra involved in cell surface modification

Locus ID	Gene	Product	AOA	COG	COG class	COG function	Pfam	Pfam function	Pfam e-value
Cluster 1									
NDEV_0129	_	Putative glycosyltransferase	Y	COG1807	M	4-amino-4-deoxy-L-arabinose transferase and related glycosyltransferases of PMT family	-	-	-
NDEV_0130	cdc	Cell division control protein 6 family protein	Y	COG1474	LO	Cdc6-related protein, AAA superfamily ATPase	PF09079	CDC6, C-terminal domain	9.00E-09
NDEV_0131	_	Putative glucose-6-phosphate/mannose-6-phosphate isomerase	Y	COG0166	G	Glucose-6-phosphate isomerase	PF10432	Bifunctional glucose-6-phosphate/mannose-6-phosphate isomerase	9.90E-36
NDEV_0132	capD1	Polysaccharide biosynthesis protein CapD	N	COG1086	MG	Predicted nucleoside-diphosphate sugar epimerases	PF02719	Polysaccharide biosynthesis protein CapD-like	1.20E-87
NDEV_0133	spsC1	Pyridoxal phosphate-dependent aminotransferase, cell wall synthesis	N	COG0399	M	Predicted pyridoxal phosphate-dependent enzyme apparently involved in regulation of cell wall biogenesis	PF01041	DegT/DnrJ/EryC1/StrS aminotransferase	3.00E-103
NDEV_0134	_	Putative glycosyltransferase	N	COG0438	M	Glycosyltransferase	PF00534	Glycosyl transferase, family 1	5.80E-30
NDEV_0135	_	Putative SAM-dependent methyltransferase	N	COG0500	QR	SAM-dependent methyltransferases	PF02911	Formyl transferase, C-terminal	0.00016
							PF13489	Methyltransferase domain	1.00E-12
NDEV_0136	_	Putative glycosyltransferase	N	COG1887	M	Putative glycosyl/glycerophosphate transferases involved in teichoic acid biosynthesis TagF/TagB/EpsJ/RodC	PF04464	CDP-glycerol glycerophosphotransferase	4.00E-13
NDEV_0137	spsC2	Putative pyridoxal phosphate-dependent aminotransferase	Y	COG0399	M	Predicted pyridoxal phosphate-dependent enzyme apparently involved in regulation of cell wall biogenesis	PF01041	DegT/DnrJ/EryC1/StrS aminotransferase	1.10E-90
NDEV_0138	_	GDP-mannose mannosyl hydrolase	Y	COG1051	F	ADP-ribose pyrophosphatase	PF00293	NUDIX hydrolase domain	8.00E-07
NDEV_0139	gca	GDP-mannose 4,6-dehydratase	N	COG1089	M	GDP-D-mannose dehydratase	PF01370	NAD-dependent epimerase/dehydratase	1.60E-42
NDEV_0140	_	Putative methyltransferase	N	COG0500	QR	SAM-dependent methyltransferases	PF08484	C-methyltransferase	1.30E-44
							PF08421	Methyltransferase putative zinc binding domain	5.00E-12

							PF13489	Methyltransferase domain	4.30E-13
NDEV_0141	_	Putative sulfotransferase	N	-	-	-	PF13469	Sulfotransferase, family 3	6.20E-10
NDEV_0142	cysC	Putative adenylyl-sulfate kinase	N	COG0529	P	Adenylylsulfate kinase and related kinases	PF01583	Adenylylsulphate kinase	2.70E-50
NDEV_0143	_	Putative glycosyl transferase	N	COG0463	M	Glycosyltransferases involved in cell wall biogenesis	PF00535	Glycosyl transfera, family 2	1.60E-09
NDEV_0144	_	Putative glycosyl/glycerophosphate transferase	N	COG1887	M	Putative glycosyl/glycerophosphate transferases involved in teichoic acid biosynthesis TagF/TagB/EpsJ/RodC	PF04464	CDP-glycerol glycerophosphotransferase	6.50E-14
NDEV_0145	_	Putative sialyltransferase	N	-	-	-	-	-	-
NDEV_0146	_	Putative LpxA family acetyltransferase	N	COG0110	R	Acetyltransferase (isoleucine patch superfamily)	PF00132	Bacterial transferase hexapeptide repeat	1.80E-08
NDEV_0147	neuC	UDP-N-acetylglucosamine 2-epimerase	N	COG0381	M	UDP-N-acetylglucosamine 2-epimerase	PF02350	UDP-N-acetylglucosamine 2-epimerase	4.30E-85
NDEV_0148	hemL	Glutamate-1-semialdehyde aminotransferase	Y	COG0001	H	Glutamate-1-semialdehyde aminotransferase	PF00202	Aminotransferase class-III	9.10E-42
NDEV_0149	_	Oxidoreductase-like protein	Y	COG0673	R	Predicted dehydrogenases and related proteins	PF01408	Oxidoreductase family, NAD-binding	3.2E-13
NDEV_0150	neuB1	N-acetylneuraminate synthase	N	COG2089	M	Sialic acid synthase	PF03102	N-acetylneuraminic acid synthase	6.70E-84
NDEV_0151	kpsU1	Acylneuraminate cytidylyltransferase	N	COG1861	M	Spore coat polysaccharide biosynthesis protein F, CMP-KDO synthetase homolog	PF02348	Acylneuraminate cytidylyltransferase	1.4E-12
NDEV_0152	neuB2	N-acetylneuraminate synthase	N	COG2089	M	Sialic acid synthase	PF03102	N-acetylneuraminic acid synthase	1.30E-45
NDEV_0153	_	SAM-dependent methyltransferase	N	COG0500	QR	SAM-dependent methyltransferases	PF13489	Methyltransferase domain	6.8E-13
NDEV_0154	kpsT	Capsular polysaccharide export system, ATPase component	N	COG1134	GM	ABC-type polysaccharide/polyol phosphate transport system, ATPase component	PF00005	ABC transporter like	2.7E-08
NDEV_0155	kpsM	Capsular polysaccharide export system, permease component	N	COG1682	GM	ABC-type polysaccharide/polyol phosphate export systems, permease component	PF01061	ABC-2 type transporter	2.5E-15
NDEV_0156	_	Protein of unknown function	N	-	-	-	-	-	-
NDEV_0157	_	Putative glycosyltransferase	Y	COG0438	M	Glycosyltransferase	PF00534	Glycosyl transferase, family 1	5.80E-43
							PF13579	Glycosyl transferase 4-like domain	1.10E-16
NDEV_0158	_	Glycosyltransferase, cell wall synthesis-related	Y	COG0463	M	Glycosyltransferases involved in cell wall biogenesis	PF00535	Glycosyl transferase, family 2	5.30E-27
NDEV_0159	slp1	Putative S-layer protein	Y	-	-	-	-	-	-
Cluster 2									
NDEV_0197	_	Glycosyltransferase, group 1	Y	COG0438	M	Glycosyltransferase	PF00534	Glycosyl transferase, family 1	7.50E-33
NDEV_0198	wecB1	UDP-N-acetylglucosamine 2-epimerase	Y	COG0381	M	UDP-N-acetylglucosamine 2-epimerase	PF02350	UDP-N-acetylglucosamine 2-epimerase	2.30E-99
NDEV_0199	_	Putative glycosidase	Y	-	-	-	-	-	-
NDEV_0200	wecC	UDP-N-acetyl-D-mannosaminuronate dehydrogenase	Y	COG0677	M	UDP-N-acetyl-D-mannosaminuronate dehydrogenase	PF03720	UDP-glucose/GDP-mannose dehydrogenase	5.10E-25

							PF00984	UDP-glucose/GDP-mannose dehydrogenase	1.30E-26
NDEV_0201	_	NAD-dependent epimerase/dehydratase	Y	COG0451	MG	Nucleoside-diphosphate-sugar epimerases	PF01370	NAD-dependent epimerase/dehydratase	8.60E-62
NDEV_0202	_	Putative glycosyltransferase	Y	COG0438	M	Glycosyltransferase	PF00534	Glycosyl transferase, family 1	1.00E-18
							PF13439	Glycosyl transferase 4-like domain	6.3E-08
NDEV_0203	_	Conserved protein of unknown function	Y	-	-	-	-	-	-
NDEV_0204	_	Short-chain dehydrogenase/reductase SDR	Y	COG1028	IQR	Dehydrogenases with different specificities (related to short-chain alcohol dehydrogenases)	PF13561	Enoyl-(Acyl carrier protein) reductase	6.5E-07
NDEV_0205	wecB2	UDP-N-acetylglucosamine 2-epimerase	Y	COG0381	M	UDP-N-acetylglucosamine 2-epimerase	PF02350	UDP-N-acetylglucosamine 2-epimerase	4.50E-88
NDEV_0206	_	Putative glycosyltransferase	Y	COG0438	M	Glycosyltransferase	PF00534	Glycosyl transferase, family 1	6.00E-30
							PF13477	Glycosyl transferase 4-like domain	4.90E-05
NDEV_0207	_	Putative sulfatase	Y	-	-	-	PF00844	Sulfatase	7.3E-06
NDEV_0208	spsC3	Pyridoxal phosphate-dependent aminotransferase, cell wall synthesis	Y	COG0399	M	Predicted pyridoxal phosphate-dependent enzyme apparently involved in regulation of cell wall biogenesis	PF01041	DegT/DnrJ/EryC1/StrS aminotransferase	1.40E-98
NDEV_0209	_	Putative aminoglycoside phosphotransferase	N	COG2334	R	Putative homoserine kinase type II (protein kinase fold)	PF01636	Aminoglycoside phosphotransferase	7.3E-12
NDEV_0210	neuB3	N-acetylneuraminate synthase	N	COG2089	M	Sialic acid synthase	PF03102	N-acetylneuraminic acid synthase	3.30E-56
NDEV_0211	kpsU2	Acylneuraminate cytidylyltransferase	N	COG1861	M	Spore coat polysaccharide biosynthesis protein F, CMP-KDO synthetase homolog	PF02348	Acylneuraminate cytidylyltransferase	1.10E-17
NDEV_0212	neuB4	N-acetylneuraminate synthase	N	COG2089	M	Sialic acid synthase	PF03102	N-acetylneuraminic acid synthase	1.90E-70
NDEV_0213	_	Putative LpxA family acetyltransferase	N	COG0110	R	Acetyltransferase (isoleucine patch superfamily)	PF00132	Bacterial transferase hexapeptide repeat	1.6E-06
NDEV_0214	_	Conserved protein of unknown function	Y	-	-	-	-	-	-
NDEV_0215	_	Putative nucleoside-diphosphate-sugar epimerase	N	COG0451	MG	Nucleoside-diphosphate-sugar epimerases	-	-	-
NDEV_0216	_	Putative phytanyol-CoA dioxygenase	N	-	-	-	PF05721	Phytanoyl-CoA dioxygenase	1.2E-09
NDEV_0217	_	Putative uncharacterised deacetylase, LmbE-like	N	COG2120	S	Uncharacterized proteins, LmbE homologs	PF02585	N-acetylglucosaminyl phosphatidylinositol deacetylase	2.10E-25
NDEV_0218	_	WbqC-like family protein	N	-	-	-	PF08889	WbqC-like protein family	1.00E-69
NDEV_0219	spsG	Putative pseudaminic acid biosynthesis-associated protein	N	COG3980	M	Spore coat polysaccharide biosynthesis protein, predicted glycosyltransferase	PF04101	Glycosyl transferase, family 28	5.5E-09
NDEV_0220	capD2	Polysaccharide biosynthesis protein CapD	Y	COG1086	M	Predicted nucleoside-diphosphate sugar epimerases	PF02719	Polysaccharide biosynthesis protein CapD-like	3.30E-89
NDEV_0221	_	Putative glycosyl/glycerophosphate transferase	Y	COG1887	M	Putative glycosyl/glycerophosphate transferases involved in teichoic acid biosynthesis TagF/TagB/EpsJ/RodC	PF04464	CDP-glycerol glycerophosphotransferase	2.90E-22

NDEV_0222	_	Putative surface antigen transporter	Y	COG2244	R	Membrane protein involved in the export of O-antigen and teichoic acid	PF13444	Polysaccharide biosynthesis protein	4.2E-06
NDEV_0223	_	Putative glycosyltransferase	Y	COG0438	M	Glycosyltransferase	PF00534	Glycosyl transferase, family 1	1.1E-08
							PF13477	Glycosyl transferase 4-like domain	2.1E-08
NDEV_0224	_	Putative acyl-CoA N-acyltransferase	Y	-	-	-	PF02388	FemAB family protein	8.8E-07
NDEV_0225	_	Conserved protein of unknown function	N	-	-	-	-	-	-
NDEV_0226	spsC4	Putative pyridoxal phosphate-dependent aminotransferase	N	COG0399	M	Predicted pyridoxal phosphate-dependent enzyme apparently involved in regulation of cell wall biogenesis	PF01041	DegT/DnrJ/EryC1/StrS aminotransferase	3.30E-17
NDEV_0227	_	NAD-dependent epimerase/dehydratase	N	COG0451	MG	Nucleoside-diphosphate-sugar epimerases	PF01370	NAD-dependent epimerase/dehydratase	2.90E-52
NDEV_0228	spsC5	Putative pyridoxal-phosphate dependent aminotransferase	Y	COG0399	M	Predicted pyridoxal phosphate-dependent enzyme apparently involved in regulation of cell wall biogenesis	PF01041	DegT/DnrJ/EryC1/StrS aminotransferase	5.40E-87
NDEV_0229	_	Putative UDP-glucose/GDP-mannose dehydrogenase	Y	COG0028	EH	Thiamine pyrophosphate-requiring enzymes	-	-	-
NDEV_0230	_	NAD-dependent epimerase/dehydratase	Y	COG0451	MG	Nucleoside-diphosphate-sugar epimerases	PF01370	NAD-dependent epimerase/dehydratase	3.60E-63

Supplementary Table 9. Characterised ammonium/ammonia transporters in published literature

Organism	Gene	Limiting substrate	Evidence	Supplementary reference
Amt family transporters				
Escherichia coli	*AmtB*	Ammonium*	X-ray crystallography/intracellular pH measurement	2
Corynebacterium glutamicum	*Amt*	Ammonium	Effect of pH on uptake	3,4
Archaeoglobus fulgidus	*AfAmt-1*	Ammonium	Modelling based on free energy calculations;	5
			X-ray crystallography**	6
Triticum aestivum (wheat)	*TaAMT1;1*	Ammonium	Electrophysiology/Effect of pH on uptake	7
Saccharomyces cerevisiae	*Mep1-3*	Ammonium	Effect of pH on uptake	8
Lycopersicon esculentum (tomato)	*LeAMT1;1*	Ammonium	Electrophysiology	9,10
	LeAMT1;2	Ammonium	Electrophysiology/Effect of pH on uptake	11
Phaseolus vulgaris (bean)	*PvAMT1;1*	Ammonium	Electrophysiology/Effect of pH on uptake	12
Arabidopsis thaliana	*AtAMT1;1*	Ammonium	Electrophysiology/Effect of pH on uptake	13
	AtAMT2	Ammonium	Effect of pH on uptake	14
Lotus japonicus	*JpAMT2;2*	Ammonium	Electrophysiology/Effect of pH on uptake	15
Rh family transporters				
Homo sapiens (human)	*RhAG*	Ammonia	Effect of pH on uptake	16
	RhBG	Ammonia	Electrophysiology/Effect of pH on uptake	17
	RhCG	Ammonia	Intracellular pH measurement;	18
			Electrophysiology/Effect of pH on uptake	19
Nitrosomonas europaea	*Rh*	Ammonia	X-ray crystallography;	20
			Effect of pH on uptake	21

*Although Amt transporters have been reported to bind ammonium (and therefore be restricted by ammonium, rather than ammonia, concentration), there is evidence for several distinct mechanisms for transport, including uniport of NH_4^+, binding of NH_4^+ followed by transport of NH_3 and co-transport of NH_3 and H^+.

**X-ray crystallography of AfAmt-1 could not resolve whether ammonium is the bound substrate

Supplementary Table 10. Regression analysis of transcript abundance and NO_2^- production during pH shock

	pH					
	4		5		6	
	p-value	r^2	*p*-value	r^2	*p*-value	r^2
amoA	0.67	0.01	<0.01*	0.42	<0.01*	0.68
amt1a	0.26	0.11	0.19	0.13	0.62	0.04
amt1b	0.59	0.02	0.63	0.02	0.04*	0.33
amt2	0.09	0.21	0.02*	0.34	<0.01*	0.79
kdpB	0.77	0.01	0.34	0.08	<0.01*	0.53
cam	0.02*	0.34	0.70	0.01	0.55	0.03
alsD	0.16	0.21	0.71	0.02	0.40	0.24
Nitrite	<0.01*	0.54	<0.01*	0.99	<0.01*	0.46

Supplementary references

1. Käll L, Krogh A, Sonnhammer ELL. 2004. A Combined Transmembrane Topology and Signal Peptide Prediction Method. J Mol Biol **338**:1027-36.
2. Khademi S, O'Connell J, Remis J, Robles-Colmenares Y, Miercke LJ, Stroud RM. 2004. Mechanism of ammonia transport by Amt/MEP/Rh: structure of AmtB at 1.35 A. Science **305**:1587-94.
3. Meier-Wagner J, Nolden L, Jakoby M, Siewe R, Krämer R, Burkovski A. 2001. Multiplicity of ammonium uptake systems in *Corynebacterium glutamicum:* role of Amt and AmtB. Microbiology **147**:135–143.
4. Siewe RM, Weil B, Burkovski A, Eikmanns BJ, Eikmanns M, Kramer R. 1996. Functional and Genetic Characterization of the (Methyl)ammonium Uptake Carrier of *Corynebacterium glutamicum.* J Biol Chem **271**:5398-5403.
5. Ullmann RT, Andrade SL, Ullmann GM. 2012. Thermodynamics of transport through the ammonium transporter Amt-1 investigated with free energy calculations. J Phys Chem B **116**:9690-703.
6. Andrade SLA, Dickmanns A, Ficner R, Einsle O. 2005. Crystal structure of the archaeal ammonium transporter Amt-1 from *Archaeoglobus fulgidus*. *Proc Natl Acad Sci USA* **102:** 14994–14999.
7. Søgaard R, Alsterfjord M, MacAulay N, Zeuthen T. 2009. Ammonium ion transport by the AMT/Rh homolog TaAMT1;1 is stimulated by acidic pH. Pflugers Arch - Eur J Physiol **458**: 733–743.
8. Boeckstaens M, André B, Marini AM. 2008. Distinct Transport Mechanisms in Yeast Ammonium Transport/Sensor Proteins of the Mep/Amt/Rh Family and Impact on Filamentation. J Biol Chem **283**:21362-21370.

9. Ludewig U, von Wirén N, Frommer WB. 2002. Uniport of NH by the Root Hair Plasma Membrane Ammonium Transporter LeAMT1;1. J Biol Chem **277**:13548-13555.

10. Mayer M, Dynowski M, Ludewig U. 2006. Ammonium ion transport by the AMT/Rh homologue LeAMT1;1. Biochem J **396**:431-7.

11. Ludewig U, Wilken S, Wu B, Jost W, Obrdlik P, El Bakkoury M, Marini AM, Andre B, Hamacher T, Boles E, von Wiren N, Frommer B. 2003. Homo- and Hetero-oligomerization of Ammonium Transporter-1 NH_4^+ Uniporters. J Biol Chem **278**:45603-45610.

12. Ortiz-Ramirez C, Mora SI, Trejo J, Pantoja O. 2011. PvAMT1;1, a Highly Selective Ammonium Transporter That Functions as H^+/NH_4^+ Symporter. J Biol Chem **286**:31113-31122.

13. Wood CC, Poree F, Dreyer I, Koehler GJ, Udvardi MK. 2006. Mechanisms of ammonium transport, accumulation, and retention in oocytes and yeast cells expressing *Arabidopsis* AtAMT1;1. FEBS Letters **580**:3931–3936.

14. Sohlenkamp C, Wood CC, Roeb GW, Udvardi MK. 2002. Characterization of *Arabidopsis* AtAMT2, a high-affinity ammonium transporter of the plasma membrane. Plant Physiol **130**:1788-96.

15. Guether M, Neuhäuser B, Balestrini R, Dynowski M, Ludewig U. 2009. A Mycorrhizal-Specific Ammonium Transporter from Lotus japonicus Acquires Nitrogen Released by Arbuscular Mycorrhizal Fungi. Plant Physiol **150**:73–83.

16. Westhoff CM, Ferreri-Jacobia M, Mak DD, Foskett JK. 2002. Identification of the Erythrocyte Rh Blood Group Glycoprotein as a Mammalian Ammonium Transporter. J Biol Chem **277**:12499-12502.

17. Ludewig U. 2004. Electroneutral ammonium transport by basolateral rhesus B glycoprotein. J Physiol **559**: 751-759.

18. Ripoche P, Bertrand O, Gane P, Birkenmeier C, Colin Y, Cartron JP. 2004. Human Rhesus-associated glycoprotein mediates facilitated transport of NH3 into red blood cells. *Proc Natl Acad Sci USA* **101**:17222–17227.

19. Mayer M, Schaaf G, Mouro I, Lopez C, Colin Y, Neumann P, Cartron JP, Ludewig U. 2006. Different transport mechanisms in plant and human AMT/Rh-type ammonium transporters. J Gen Physiol. **127**:133-44.

20. Li X, Jayachandran S, Nguyen HT, Chan MK. 2007. Structure of the *Nitrosomonas europaea* Rh protein. *Proc Natl Acad Sci USA* **104**:19279-84.

21. Weidinger K, Neuhäuser B, Gilch S, Ludewig U, Meyer O, Schmidt I. 2007. Functional and physiological evidence for a rhesus-type ammonia transporter in *Nitrosomonas europaea.* FEMS Microbiol Lett **273**:260-7.
